# Determinants of Household Solid Waste Generation and Composition in Homs City, Syria

**DOI:** 10.1155/2020/7460356

**Published:** 2020-11-26

**Authors:** Mohamad Noufal, Liu Yuanyuan, Zena Maalla, Sylvia Adipah

**Affiliations:** ^1^College of Environment and Ecology, Chongqing University, Chongqing City, China; ^2^Faculty of Civil Engineering, Al-Baath University, Homs City, Syria; ^3^College of Earth Environmental and Sciences, Lanzhou University, Lanzhou City, China

## Abstract

The absence of accurate information on the state of waste is a challenge to the solid waste management system in Syria. The local authorities commonly estimate the quantity of waste produced and its characterisation, which is the starting point for solid waste management planning. So, this paper aims to evaluate the generation and composition of household solid waste in Homs city, Syria. Also, the study presents factors influencing the waste generation rate and the waste composition. The study was carried out in 300 families from four zones in Homs city, and three sampling stages were conducted during the study duration, which started in July 2017 and ended in February 2019. The outcomes show that an average of 0.68 kg/per/day solid waste generated was calculated for the entire study area in Homs city. Also, the data analysis presents that organic waste constitutes the largest component in the waste mixture (69.1%) followed by plastic (10.6%), inert materials (8.7%), paper (4.6%), textile (2.5%), metal (1.2%), glass (1.1%), wood (0.6%), and hazardous materials (1.6%). The multiple linear regression results showed that the adjusted *R*^2^ value was found to be 0.557, 0.839, and 0.709 for the waste generation per capita, the daily household organic waste generation, and the daily household packaging waste generation, respectively. Also, according to Pearson's coefficient values, a positive correlation was found between household waste generation and monthly income (*r* = 0.626), household size (*r* = 0.37), and age of the household head (*r* = 0.517), whereas a negative correlation was found between household waste generation and the education level of the household head (*r* = −0.649).

## 1. Introduction

Solid waste problem is a significant concern for national and local authorities in many cities of developing countries [1]. The poor conditions of municipal solid waste (MSW) in these areas are represented by the accumulation of waste in the streets, a low waste collection rate, and the random dumping or burning trash in open spaces [2–5]. Improper waste disposal practices such as burning or dumping of waste at roadsides and vacant lands may cause environmental, health, and aesthetic damage, as well as depletion of natural and economic resources [6–12].

At first and before making decisions regarding improving the current waste practices or proposing new waste management scenarios, it is significant to have an overall perception of various aspects related to waste issues. The first and fundamental point is to know the amount and characteristics of waste generated in order to determine the most successful and efficient waste management plans [13–21]. Waste generation rates and composition differ from one nation to another and even between cities within a country since they are influenced by factors such as the level of industrialisation, the climate, and the nature of socioeconomic development [21]. It is common in developing countries that the daily amount of waste collected is not equal to the amount of actual waste produced by households. This can be attributed to inadequate waste collection services and informal waste picking activities. Thus, basic data on waste characteristics that are indispensable for the design and planning of the solid waste management facilities are absent [22], or at best, they are ambiguous and untrustworthy as they are derived from different sources based on estimates and judgments rather than correct measurements and field investigations [23].

Several research studies have been conducted to investigate the generation and composition of household solid waste in various regions over the world [24–35]. These studies indicated that waste characteristic study is critical for a few reasons, for example, the necessity to identify the potential of material recuperation from the waste mixture, to determine waste generation sources, and to ease the planning of treatment facilities.

Furthermore, household solid waste is highly heterogeneous and is widely dependent on the socioeconomic status of the households [19, 36]. The combination of socioeconomic factors identifies how a social hierarchy is organised, one's position within this structure [37]. Although the features of urban areas in developing countries are common, waste management strategies should respond to local conditions and be inventive, decisive, and context-sensitive [9, 14]. Environment issues related to waste generation are part of societal changes where households play a significant role. These societal changes impact the characteristic of given households, including family size, monthly income, social status, education level, residential location, and community status. Many studies have been carried out to illustrate the relationship between socioeconomic factors of households and solid waste generation and composition [24, 30, 31, 38–43].

Syria is one of the countries lacking updated and reliable data on the composition and generation of household solid waste. Moreover, SWM issue and its adverse environmental impacts were present in Syria even before the conflict began [44]. In this context, around 80% of the domestic solid waste was disposed of at illegal dumpsites, which were situated in the surrounding areas of cities. Due to the damage of waste management infrastructure, a massive amount of solid waste has accumulated in the roads within the Syrian cities [45]. In many cases, the local authorities have resorted to alternative methods such as outdoor burning or illegal dumping [46]. It is worth mentioning that no research studies were undertaken to create accurate data on the characteristics of household waste generated as well as factors influencing waste generation trends in Syrian cities. So, this paper aims to evaluate the generation and composition of household solid waste and explores factors influencing waste generation rate and composition in order to propose new postwar strategies for solid waste management in Homs city.

## 2. Materials and Methods

### 2.1. Research Area Discretion

**Figure 1:**
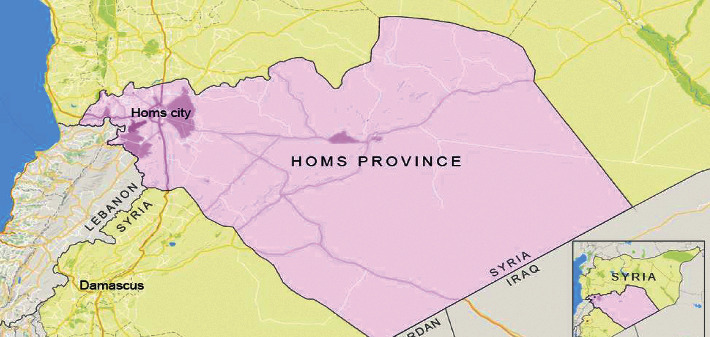
The geographical location of Homs city [49].

The city of Homs is the third biggest city in Syria, after Damascus and Aleppo, where the population before the crisis was about 800,000 [47]. Homs city occupies a central and strategical location in the country's transportation network, links all major urban centres, and besides, it is an important industrial centre. Homs city is located in central-western Syria on the Orontes river about 192 km north of the capital Damascus and 96 km inland from the Mediterranean Sea, on a plateau 501 meters above sea level. The Mediterranean Sea climate generally prevails in Homs. This climate may be characterised by rainy winter and a hot-dry summer separated by two short transitional seasons [48]. The city was one of the major urban centres to be affected by the crisis like many Syrian cities. There is a significant scale impact of the ongoing crisis on the city's housing, economy, infrastructure, and services. As it usually happens in times of war, citizens from hot and unsafe areas were compelled to displace to safe districts of the city, and the same situation was in other cities. The sudden increase in the population in the safe areas of the city led to a high generation of waste which was not only a health issue but also an environmental concern to the authorities [47].  Figure 1 shows the geographical location and administrative division of Homs city.

The study targeted households residing in 4 zones (areas were relatively safe during the study period) in Homs city, as shown in Table 1.

### 2.2. Methodology

The authors used direct waste analysis as a methodology to determine waste characteristics, and this method includes sampling, sorting, and weighing the components of the waste stream [50]. The study was carried out in 300 families, which were chosen using the stratified random sampling method [51] from four zones in Homs city. The primary phase of the investigation is the sampling phase and the waste weighing, which started in July 2017 and ended in February 2019. The information from the household respondents was obtained by weighing of household waste (house-to-house) for 14 consecutive days. The sampling phase was carried out at the same time in all target zones, with a sum of three sampling stages conducted during the study duration. The first sampling stage was done in July 2017, the second stage was held in August 2018, and the last phase occurred in January and February 2019, with each period comprising 14 successive days.

The house-to-house household waste weighing procedures were carried out by four teams (each team is responsible for one zone), and each team consisted of 3 individuals. The teams utilised electronic weighing scales (10 kg maximum capacity and *α* = 10 gr reading). To determine the amount and composition of household waste, collecting waste at the generation site and directly hand sorting method was adopted, which is known to be the most accurate method for reliable data collection [52]. The responsibilities of teams were to recognise and take the waste bag of responding households and then weigh these materials, which were weighed and sorted into particular categories, for example, metals, plastics, glass, and paper.

The face-to-face technique was employed because it appeared to be the most reliable way of a questionnaire. The questionnaire was completed in August 2017, which continued for 25 days. The survey comprised inquiries relating to the demographic conditions, socioeconomic situation, and the practices of domestic waste management propensities at the household level in the studied areas. As a result, data were gathered on a wide range of subjects such as personal and household characteristics, income, wealth, access to infrastructure, and attitudes towards the environment. Hence, information on waste generation was collected and analysed with the objective to identify the waste generation rate per capita. Also, the socioeconomic information obtained from the survey was studied to understand the relation between the socioeconomic conditions of the respondents and the waste generation rate.

### 2.3. Empirical Model for Household Solid Waste Generation and Composition

Notwithstanding the quantification of household solid waste, it is basic to model waste generation in order to identify the factors that influence the waste generation rate of the households selected and design policies to lessen it. The correlation analysis was employed to comprehend the independent variables that predict the quantity of waste generated. Then, linear regression analysis was used to find the relationship between waste generation and socioeconomic variables. The current research uses ordinary least squares as a multiple linear regression model which is the most commonly used technique for parameter estimation due to its simplicity [53, 54]. The model can be expressed as

(1) $$\begin{array}{c} y_{i}=\beta_{0}+x_{i}*\beta_{i}+\varepsilon_{i}, \end{array}$$

where

 
*y*_*i*_ = waste generation per capita/waste composition (dependent variable)
 
*x*_*i*_ = independent variables
 
*i* = number of observations
 
*β*_0_ = constant term
 
*β*_*i*_ = coefficient of independent variables
 
*ε* = the error or disturbance term

In this regression analysis, waste generation per capita is regressed quantitatively by several independent variables. The independent variables are household size, monthly income, education level of the household head, age of the household head, and the gender of the household head. Empirical specification for the model can be explained by

(2) $$\begin{array}{c} \text{waste generation}\left(\text{per capita}\right)=\beta_{0}+\beta_{1}\left(\text{household size}\right)+\beta_{2}\left(\text{monthly income}\right)+\beta_{3}\left(\text{education level}\right)+\beta_{4}\left(\text{gender}\right)+\beta_{5}\left(\text{age}\right)+\varepsilon . \end{array}$$

### 2.4. Data Analysis

Data obtained from the waste characteristics and data generated from the questionnaire survey study were analysed using Microsoft Excel for Windows (Excel) and Statistical Package for the Social Sciences (SPSS). Some of the data from the questionnaire survey were nominal in nature. As per [55, 56], such data are best analysed using inferential (correlation and regression models) and descriptive statistics. In the beginning, data were subjected to a test for normality which showed that data were approximately normally distributed. Then, descriptive statistics were assembled as the variables were nominal and ordinal. Analysis of such rating data was done using parametric statistical tests, namely, analysis of variance (ANOVA).

Regression analysis was employed to assess the linear relationships for studied variables to identify the major factors impacting the waste generation rate. The Pearson correlation coefficient was utilised in correlation analysis before regression analysis to understand the relationship between the rate of waste generation and socioeconomic characteristics of households. Per capita waste generation and waste composition were considered as the dependent variables in the developed regression models. The socioeconomic characteristics of households (household size, monthly income, gender of the household head, education level of the household head, and age of the household head) were considered as independent variables. In regression models, standard criteria, *R*^2^, *F*, and *t*-tests, were used to test the significance of outcomes. The *R*^2^ value is the squared multiple correlation coefficients which represent the correlation between dependent and independent variables. In order to explain the predictive capacity of the regression models, *F*-test was used. The *t*-statistics were used to examine the significance of the correlation coefficients of the independent variables (*β*) estimated.

## 3. Results and Discussion

### 3.1. Socioeconomic Characteristics of the Target Households

The characteristics of the sample studied are illustrated in Table 2. The gender distribution of the household head in the sample was found to be 179 (59.67%) males and 121 (40.33%) women. Concerning the age of the household head, 43 (14.30%) participants aged between 18 and 30 years, 100 (33.33%) respondents were between 31 and 45 years, 122 (40.70%) respondents were between 46 and 60 years, and the remaining 35 (11.67%) respondents were more than 60 years. With regard to family size, 44 (14.70%) households consisted of 1–3 people, 135 (45.00%) households consisted of 4–6 people, and 121 (40.30%) households included more than 6 people. With regard to monthly income, only 9 respondents (3%) earned less than 50,000 SYP per month, 135 respondents (45%) earned between 50,001 and 100,000 SYP per month, and 108 respondents (36%) earned between 100,001 and 150,000 SYP per month. On the contrary, 108 respondents (14%) earned between 150,001 and 200,000 SYP per month, and the remaining 6 respondents (2%) earned more than 200,000 SYP. Regarding the education level of the household head, 19 (6.33%) respondents had elementary education, 34 (11.33%) respondents went to middle schools, and 100 (33.33%) respondents obtained certificate from high school. On the contrary, 131 (43.00%) respondents received education at the university or institute level, and 16 (5.00%) participants completed the postgraduate level.

### 3.2. Average Daily Generation per Capita

The per capita waste generation is calculated by dividing the total waste generated with the number of people living in that household that day [58]. According to results, 0.68 kg/person/day was calculated for the entire study area in Homs city. As it is shown in Table 3, zone 4 had the highest waste generation rate per capita daily (0.74 kg/capita/day), and zone 1 had the lowest waste generation rate per capita daily (0.61 kg/capita/day).

ANOVA test results in Table 4 show that there is a significant difference in the waste generation per capita across the four sampling zones.

According to the literature, the results of per capita daily waste generation studies for various cities in developing countries were 0.12 kg/per/day in Oyo city, Nigeria [59], 0.21 kg/per/day for Cape Haitian city in the Republic of Haiti [30], 0.25 kg/per/day for Chittagong in Bangladesh [38], 0.28 kg/per/day for Mekong Delta city in Vietnam [32], 0.34 kg/per/day for Olongapo city in the Philippines [60], 0.49 kg/per/day for Kathmandu in Nepal [58], 0.53 kg/per/day for urban areas in Bhutan [61], 0.62 kg/cap/day in Mostaganem city, Western Algeria [62], 0.634 kg/cap/day in Abuja city, Nigeria [63], 0.67 kg/per/day for Chihuahua in Mexico [64], and 0.82 kg/per/day in Nablus city, Palestine [33]. On the contrary, the results of per capita daily waste generation studies in European countries were 2.12 kg/per/day in Denmark, 1.75 kg/per/day in Norway, 1.56 kg/per/day in France, 1.4 kg/per/day in the Netherlands, and 1.28 kg/per/day in the United Kingdom [65]. Various values of per capita daily waste generation can be attributed to levels of urbanisation, lifestyles, and many other factors specific to particular areas.

### 3.3. Waste Composition

The waste composition provides an insight that helps to improve the sustainability of waste management as the quantity and methods of solid waste diverted from landfills mainly depend on the composition of waste. Moreover, this aids in identifying the recycling possibilities to justify the need for recyclable waste collection services and to determine a charging method for mixed waste to encourage waste recycling programs. Waste composition analysis showed that most of the waste generated were organic wastes (69.1%) followed by plastic (10.6%), inert materials (8.7%), paper (4.6%), textile (2.5%), metal (1.2%), glass (1.1%), wood (0.6%), and hazardous materials (1.6%).

As shown in Table 5, the portion of food and kitchen waste (organic part) found in the study has always been more than 2/3 of the total composition of waste in all selected zones. The high percentage of organic content in the household solid waste generated from Homs neighbourhoods can be attributed to the fact that Syria's economy is agro-based, and there is a high level of consumption. The high content of organic fraction can be used for composting as a possible method to diminish the quantity of waste that needs to be transported to the landfill, also to convert the organic part of waste into compost. In case the waste is not collected, the organic portion of waste will be increasingly disintegrated causing the release of unpleasant odours and ground and surface water pollution through leachates and attracting insects and rodents to piles of garbage in the street.

Plastic and paper materials present a good existence in the waste stream in Homs city (10.60% and 4.60%, respectively) which causes a visual nuisance and crucial ecological issues incorporating the blockage of drainages, soil deterioration, and contamination of surface water. Inert materials which form 8.7% of the waste output in Homs city mostly arise from home and road sweeping activities, ashes, and residues of demolition and construction work. Other essential components in the waste mixture are textiles (rags, discarded clothes, and cuttings), metals (abandoned vehicles, white goods, and households' hardware), and glass (glass bottles and broken kitchen utensils). Broken glass and sharp metal piece are real perils and favourite reasons for injury, particularly for scavengers who scatter garbage pile dumps searching for saleable items and kids who play or stay close to the waste dumps.

ANOVA test results from Table 6, however, showed that the means of six material components, which are plastics, paper, textile, glass, wood, and hazardous materials, differed or varied significantly across the sampling zones, while organic waste, inert materials, and metal had no significant statistical variations across the sampling zones.

The composition of waste is different from place to place and relies on variables, for example, geographical location, climate condition, cooking and eating habits, cultural traditions, level of advancement of the nation, and socioeconomic status [66, 67]. Thus, the composition reported in our study was compared to other studies conducted in different countries (as shown in Table 7).

### 3.4. Factors That Affect Solid Waste Characteristics

#### 3.4.1. Family Size

Family size is an important component in determining the amount of household waste. In this research, family size refers to the overall people living in the same house. Previous studies [18, 20, 36–38, 59, 80–86] showed that household size had a positive influence on the waste generation rate. While it is apparent for more members of a family to generate more waste, some researchers described the phenomena of “group living” and “common consumption” of the family as the household operates as a unit and most of the food items are shared. Therefore, the fewer amount of food crumbs, leftovers, and packaging waste will be produced [40, 52]. On the contrary, many studies have also supported the negative relationship between the household size and the waste generation rate [19, 31, 32, 63, 82, 87–91].

**Figure 2:**
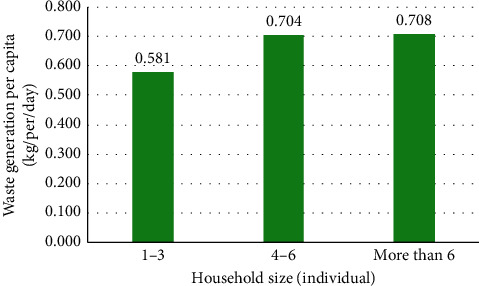
The relationship between family size and per capita waste generation.

The outcomes which are displayed in Figure 2 show that household size had a positive influence on the waste generation rate, and it is apparent for more members of a family to generate more waste. Also, a statistical method of bivariate analysis (Pearson's coefficient) was used to test the correlation between household waste generation and household size. In the present study, a medium positive correlation (*r* = 0.37, *P* < 0.01) was found between household waste generation and family size.

#### 3.4.2. Monthly Income

The affluence or income level of a household is one of the influencing factors believed to play a direct role in deciding waste generation rates and composition [92]. Medina [93] indicated that waste generation is directly linked to the income levels of households, and the higher-income members consume more products, and their waste includes more recyclable items. The increase in the income level leads to a clear difference in amounts and composition of waste generated due to changes in the pattern of households' consumption [63]. Many research studies [30, 32, 36, 38, 52, 58, 59, 63, 82–85, 87, 88, 94–98] supported the idea that the household income has a direct and positive relationship with the daily per capita waste generation. As per those studies, the higher the income of a household, the higher its purchasing power, and this can be the reason for income being a positive impact on the amount of waste. On the contrary, Qu et al. and Monavari et al. [31, 35] found that family income has a negative impact on the waste generation rate. Also, Trang et al. [20] indicated that higher-income households prefer to eat outside more frequently than cooking at home, thereby generating less waste.

**Figure 3:**
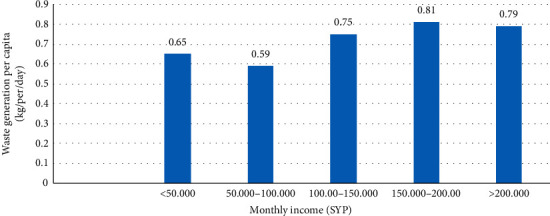
The relationship between family income and per capita waste generation.

The outcomes which are displayed in Figure 3 show that monthly income had a positive influence on the waste generation rate, and it is apparent that high-income households generate more waste. Also, a statistical method of bivariate analysis (Pearson's coefficient) was used to test the correlation between household waste generation and monthly income. In the present study, a strong positive correlation (*r* = 0.626, *P* < 0.01) was found between household waste generation and monthly income.

#### 3.4.3. Education Level

The educational status amongst the inhabitants can notably influence the prosperity of awareness programs aimed at evolving solid waste management practices. The more a family gets educated and aware of the adverse impacts of improper solid waste management, the more it recognizes the importance of effective management of solid waste [88]. Gu et al. [52], in Suzhou city/East China, Benítez et al. [99], in Mexicali/Mexico, and Monavari et al. [35], in Ahvaz city/Iran, found that the education level of the household daily manager has a negative effect on the rate of household waste generation.

On the contrary, Kayode and Omole [88] found a positive influence of educational status on the waste generation rate in Ibadan metropolis/Nigeria. Additionally, Sujauddin et al. [38] demonstrated households with a high level of education would generate more amount of household solid waste per capita in Bangladesh. Besides, Qu et al. [31] indicated that the highest rate of domestic waste generation was generated by households with advanced education, while households with secondary education had produced the lowest rate of waste generation in Beijing city/China. Usually, higher education is related with high level of awareness on environmental issues, but sometimes, it can have an opposite relation because of the cumulative nature of education that increases with the new number of graduates every year, but environmental awareness (such as impact of higher waste generation) does not increase at the same pace [100].

**Figure 4:**
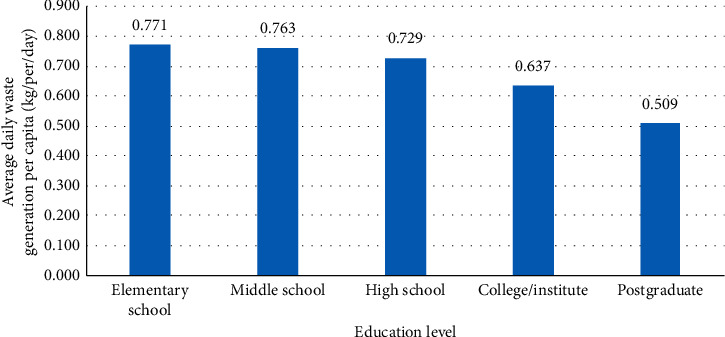
The relationship between education level and per capita waste generation.

Figure 4 demonstrates that the education level of the household head was negatively correlated with the rate of domestic solid waste generation. This means that the higher the level of education of the household member is, the lower the amount of SW produced per day. Also, a statistical method of bivariate analysis (Pearson's coefficient) was used to test the correlation between household waste generation and the education level of the household head. In the present study, a strong negative correlation (*r* = −0.649, *P* < 0.01) was found between household waste generation and the education level of the household head.

#### 3.4.4. Gender of the Household Head

**Figure 5:**
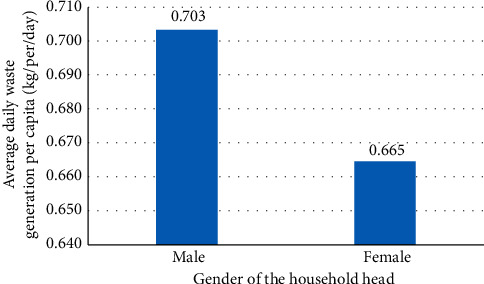
The relationship between gender and per capita waste generation.

People (male or female) may have different attitudes towards environmental issues, and hence, a gender-sensitive approach in waste management plans can promote effectiveness in resource allocation and avoid unnecessary costs. According to Kayode and Omole [88], there is an adverse effect of sex on waste generation in Nigeria. From their side, Dalen and Halvorsen [101] indicated that there are some research studies confirming women (female) producing more waste, and yet, many others do not find significant gender effects in waste generation because it is the accumulated result of all family members' behaviour.  Figure 5 demonstrates that male-headed households generate waste more than female-headed households. Also, a statistical method of bivariate analysis (Pearson's coefficient) was used to test the correlation between household waste generation and the gender of the household head. In the present study, a small positive correlation (*r* = 0.204, *P* < 0.01) was found between household waste generation and the gender of the household head.

#### 3.4.5. Age of the Household Head

The age of the household head is a continuous variable and is expected to have a correlation with the waste generation, as people may have different waste-generating behaviour according to age. Bartelings and Sterner [102] found that the elderly produce less amount of solid waste, which may be attributed to the rather modest way of life led by the aged. Furthermore, Kayode and Omole [88] noted that the age of the household head was negatively and weakly correlated with solid waste generation in Ibadan metropolis/Nigeria. Also, a study by Organisation for Economic Co-operation and Development found that middle-aged and older people are more likely to participate in waste separating and recycling programs. According to this study, the elderly are interested in and respond to social norms; so, it is likely that, with such a sense of responsibility, those people would generate lower solid waste [103]. As per Soukopová et al. [104], in the Czech Republic, the highest quantity of solid waste was generated by elder people reaching towards the end of their working career or around the time of their retirement because of different activities. Also, Talalaj and Walery [105] found that the greatest quantity of MSW is generated by the group of individuals aged 14 to 64; Beigl et al. [90] indicated that people aged 15–59 produce more waste, whilst Lebersorger and Beigl [106] found there is no significant relationship between older people and low MSW generation.

**Figure 6:**
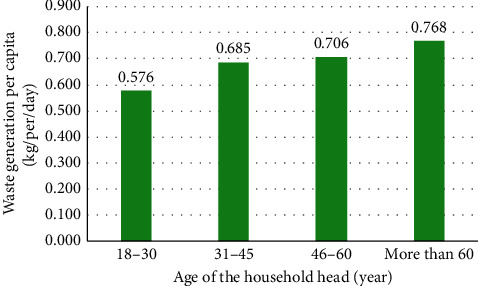
The relationship between the age of the household head and per capita waste generation.

The outcomes which are displayed in Figure 6 show that the age of the household head had a positive influence on the waste generation rate, where household waste generation increases with the age of the household head. Also, a statistical method of bivariate analysis (Pearson's coefficient) was used to test the correlation between household waste generation and the age of the household head. In the present study, a strong positive correlation (*r* = 0.517, *P* < 0.01) was found between household waste generation and the age of the household head.

#### 3.4.6. Housekeeping Activities

The research found that women are playing a significant role in waste management activities, whereas they have the essential responsibility regarding food preparation and house cleaning. Thus, they (mothers or housemaids) are directly engaged in the generation and management of household wastes. Within this context, the cleaning procedures of residential houses, which, in most of them, happen in the first part of the day (morning hours), affect the characteristics of waste as housewives mix wastes produced from house cleaning with food waste and other kinds of garbage in the same container or waste bag. This increases the weight and quantity of waste generated. The information obtained from the questionnaire and observations showed that floors and the courtyards around the houses are cleaned in the morning hours, and the trash is disposed of with other wastes in the same bags or containers. The study demonstrated that 15% of families do cleaning between 5.00 and 6.00 a.m., while 60% of households clean their houses between 7.00 and 10.00 a.m., and the rest of households (25%) demonstrated that the cleaning activities do not happen at a specific time during the day.

**Figure 7:**
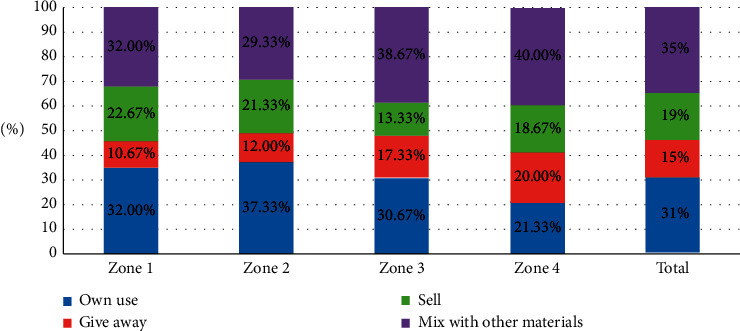
Dealing with recyclable materials in Homs neighbourhoods.

Notwithstanding, as indicated by data from the study respondents, many families retrieve glass jars and plastic bottles and by keeping them for reuse, sell, or give away to waste pickers.  Figure 7 illustrates how responding households in Homs city handle recyclable materials in the waste stream they generate. As shown in Figure 7, 31% of the respondents (93 households) kept the recyclable materials for their utilization, and 15% of the respondents (45 households) showed that they give these materials to waste pickers, while 19% of the respondents (57 households) indicated that they sell the recyclables, and finally, 35% of the respondent families (105 households) mix the recyclable materials with other materials of the waste stream in the same container or plastic bag.

A chi-square test was carried out to define the degree of association between residential districts and dealing with recyclable materials. As shown in Table 8, there is no strong statistically significant relationship between the two variables, *X*^2^ (9, *N* = 300) = 10.826, *P* > 0.05.

Waste separation at the source (at the family level) is easier, more straightforward, and effective than the separation at another level (final disposal site). Also, it reduces the separation cost and the pollution of recyclable materials that are sent to the industrial facilities. Therefore, waste separation practices should be explored at the source level. Moreover, it is difficult to detect a real situation in the study areas where families only apply the waste separation for the study period. Thus, dependence must be on the self-behaviour of families in the studied area.

### 3.5. Results of the Linear Regression Analysis

The regression model for waste generation per capita (dependent variable), along with the household size, monthly income, education level, gender, and age of the household head, is presented in Table 9. The results indicated that adjusted *R*^2^ = 0.557, meaning 55.7% of the variance of the waste generation per capita could be explained by independent variables included in the model. The independent variables in the model, namely, household size, monthly income, education level, gender, and age of the household head, were significant as suggested by the *t*-test. Moreover, the *F* value of the model suggests that the estimated model is significant (*F* = 76.262, *P* < 0.01).

According to the results, while holding other factors constant, a 1% increase in the monthly income of households contributed to a 0.04% increase in per capita waste generation, whilst a 1% increase in the number of household members contributed to a 0.009% increase in per capita waste generation. Also, a 1% increase in the age of households contributed to a 0.012% increase in per capita waste generation. On the contrary, a 1% increase in the education level of the household head resulted in a 0.035% decrease in per capita waste generation with other factors constant.

The composition of waste can also be modelled with the socioeconomic variables of the households in order to understand the relationships. Studying these relationships is useful in planning waste collection and disposal methods. The regression analysis was used to model the daily quantity of both organic and packaging wastes (paper and cardboard, plastic, glass, textile, and metal). The regression model for daily household organic waste (dependent variable), along with the household size, monthly income, education level, gender, and age of the household head, is presented in Table 10. The results indicated that adjusted *R*^2^ = 0.839, meaning 83.9% of the variance of the daily household organic waste generation could be explained by independent variables included in the model. The independent variables in the model, namely, household size, monthly income, education level, and age of the household head, were significant as suggested by the *t*-test. Moreover, the *F* value of the model suggests that the estimated model is significant (*F* = 313.338, *P* < 0.01).

According to the results, while holding other factors constant, a 1% increase in the monthly income of households contributed to a 0.098% increase in daily organic waste generation, whilst a 1% increase in the number of household members contributed to a 1.193% increase in daily organic waste generation. Also, a 1% increase in the age of households contributed to a 0.122% increase in daily organic waste generation. On the contrary, a 1% increase in the education level of the household head resulted in a 0.295% decrease in daily organic waste generation with other factors constant.

The regression model for daily household packaging waste (dependent variable), along with the household size, monthly income, education level, gender, and age of the household head, is presented in Table 11. The results indicated that adjusted *R*^2^ = 0.709, meaning 70.9% of the variance of the daily household packaging waste generation could be explained by independent variables included in the model. The independent variables in the model, namely, household size, monthly income, education level, and age of the household head, were significant as suggested by the *t*-test. Moreover, the *F* value of the model suggests that the estimated model is significant (*F* = 146.983, *P* < 0.01).

According to the results, while holding other factors constant, a 1% increase in the monthly income of households contributed to a 0.09% increase in daily packaging waste generation, whilst a 1% increase in the number of household members contributed to a 0.331% increase in daily packaging waste generation. Also, a 1% increase in the age of households contributed to a 0.031% increase in daily packaging waste generation. On the contrary, a 1% increase in the education level of the household head resulted in a 0.081% decrease in daily packaging waste generation with other factors constant.

## 4. Conclusions

The study is an initial step to understand the household waste characteristics before starting to consider introducing a new strategy for waste management in Homs city. Characterization and quantification of household wastes play a significant role in estimating material recovery potential and determining sources of generation, treatment methods, and final disposal ways. The study outcomes, which serve as a reliable database, could be helpful for the environmental planners and decision makers in their strategies for dealing with solid waste in Homs city. In this context, the dominance of the organic fraction and recyclable materials on the waste mixture in Homs city illustrates that composting and recycling are the preferred methods for handling the generated waste. Moreover, part of the waste management costs can be recovered by selling recyclables and compost, and at the same time, the challenges (public health and environmental) of uncontrolled dumping can be mitigated by lessening the amount of the waste transferred to the final disposal sites.

Like any other city in developing countries, Homs city is witnessing an increase in the amount of waste generated, but there is a lack of necessary information, infrastructure, and resources to establish an effective waste management strategy. Socioeconomic factors are key factors in behavioural studies, and the focus was on households among other groups of waste generators because, in Syrian cities, they are contributors to about 80% of the entire municipal waste generation. Looking at the socioeconomic factors, this research concluded that family size, monthly income, education level, gender of the household head, and age of the household head are significant factors to predict solid waste generation and composition trends.

It is essential to promote the involvement of local NGOs (besides the local authorities) in working on various environmental awareness programs to convince the population that the solid waste problem can be converted from an obstacle to an extra income source through separation at the source (household level). Moreover, there is an urgent need to collect accurate data on the quantities, characteristics, and types of waste generated in Syrian cities. The national government should establish a national database on waste and also support local authorities to carry out regular research studies to provide accurate data on the waste problems within their jurisdictions to facilitate waste planning and management.

## Figures and Tables

**Table 1:** Household selection for the study.

Area	Neighbourhoods	Number of participants	Family size	Residential status
Zone 1	Al Sabil, Al-Muhajirin, Al Abbasiah, Al Zahra, and Al Arman	75	6.10	Low-income areas
Zone 2	Wadi Aldahab, New Akrama, Karam el-Zeitoun, and Karam el-Looz	75	6.15	Lower-middle-income areas
Zone 3	Al Inshaat, Al-Mahatta, and Al-Shammas	75	5.95	Upper-middle-income areas
Zone 4	Al-Ghouta and Al Boughtassia	75	5.90	High-income areas

**Table 2:** Socioeconomic characteristics of the responding households.

	Frequency	Percentage (%)
Gender of the household head		
Male	179	59.67
Female	121	40.33

Age of the household head		
18–30	43	14.30
31–45	100	33.33
46–60	122	40.70
>60	35	11.67

Family size (individual)		
1–3	44	14.67
4–6	135	45.00
>6	121	40.33

Education level of the household head		
Elementary school	19	6.33
Junior high or middle school	34	11.33
High school	103	34.33
College/institute	129	43.00
Postgraduate	15	5.00

Monthly income (SYP)		
<50,000	9	3.00
50,000–100,000	135	45.00
100,000–150,000	108	36.00
150,000–200,000	42	14.00
>200,000	6	2.00

1 USD = 438 SYP [57].

**Table 3:** Average daily waste generation per capita in Homs neighbourhoods.

	Zone 1	Zone 2	Zone 3	Zone 4	Total
Waste generation per capita (kg/per/day)	0.61	0.67	0.72	0.74	0.68

**Table 4:** ANOVA test results for spatial variation in waste generation per capita.

	Sum of squares	df	Mean square	*F*	Sig.
Between groups	0.749	3	0.250	40.079	0.000
Within groups	1.844	296	0.006		
Total	2.592	299			

**Table 5:** Household solid waste composition in studied zones of Homs neighbourhoods.

	Zone 1	Zone 2	Zone 3	Zone 4	Average (%)
Organic waste (%)	72.10	71.00	66.95	66.35	69.10
Plastic (%)	8.10	9.50	12.20	12.60	10.60
Inert materials (%)	9.00	8.70	8.60	8.50	8.70
Paper (%)	3.60	4.00	5.20	5.60	4.60
Textile (%)	2.60	2.40	2.55	2.45	2.50
Metal (%)	1.30	1.20	1.15	1.15	1.20
Glass (%)	1.10	1.00	1.15	1.15	1.10
Wood (%)	0.60	0.60	0.60	0.60	0.60
Hazardous materials (%)	1.60	1.60	1.60	1.60	1.60

**Table 6:** ANOVA test results for spatial variation in waste composition.

		Sum of squares	df	Mean square	*F*	Sig.
Organic	Between groups	7.365	3	2.455	1.798	0.148
	Within groups	404.148	296	1.365		
	Total	411.513	299			

Plastic	Between groups	3.649	3	1.216	32.561	0.000
	Within groups	11.058	296	0.037		
	Total	14.707	299			

Inert materials	Between groups	0.148	3	0.049	2.263	0.081
	Within groups	6.453	296	0.022		
	Total	6.601	299			

Paper	Between groups	0.698	3	0.233	33.210	0.000
	Within groups	2.074	296	0.007		
	Total	2.772	299			

Textile	Between groups	0.016	3	0.005	3.012	0.030
	Within groups	0.536	296	0.002		
	Total	0.552	299			

Metal	Between groups	0.001	3	0.000	0.683	0.563
	Within groups	0.120	296	0.000		
	Total	0.121	299			

Glass	Between groups	0.007	3	0.002	5.808	0.001
	Within groups	0.111	296	0.000		
	Total	0.117	299			

Wood	Between groups	0.001	3	0.000	3.915	0.009
	Within groups	0.031	296	0.000		
	Total	0.032	299			

Hazardous materials	Between groups	0.009	3	0.003	3.915	0.009
	Within groups	0.221	296	0.001		
	Total	0.230	299			

**Table 7:** Waste composition (%) in developing countries.

Area	Organic	Paper	Plastic	Glass	Metal	Others	Source
Phnom Penh/Cambodia	63.3	6.4	15.5	1.2	0.6	13.0	[68]
Mekong Delta/Vietnam	80.0	4.7	6.3–7.1	0.7–1.0	0.5–0.7	0.9–1.4	[32]
Bangkok/Thailand	43.0	12.1	10.9	6.6	3.5	23.9	[69]
Bahrain/Bahrain Kingdom	59.6	9.9	13.4	5.5	3.4	9.2	[70]
Baghdad/Iraq	70.0	5.0	5.3	2.2	2.2	15.3	[71]
Amman/Jordan	54.4	14	13.2	2.8	2.4	13.2	[72]
Abadan/Iran	66.9	11.2	14.3	2.8	1.35	3.45	[73]
Chittagong/Bangladesh	62.0	3.0	2.0	5.0	9.0	3.0	[38]
Kathmandu/Nepal	71.0	7.5	12.0	1.3	0.5	7.9	[58]
Lagos/Nigeria	68.0	10.0	7.0	4.0	3.0	8	[74]
Cape Haitian/Haiti	65.5	9.0	9.2	5.8	2.6	7.9	[30]
Bhutan	62.2	15.2	13.1	2.7	0.7	6.1	[61]
Chihuahua/Mexico	48.0	16.1	11.9	5.9	2.4	16.0	[64]
Ghana	61.0	5.0	14.0	3.0	3.0	14.0	[19]
Nablus/Palestine	65.1	9.1	7.6	2.9	2.8	5.4	[33]
Moratuwa/Sri Lanka	90.0	5.0	3.0	2.0	1.0	—	[75]
Allahabad/India	45.3	4.69	2.86	0.73	2.54	43.88	[76]
Portugal	35.5	25.9	11.5	5.4	2.6	19.1	[34]
Kraków/Poland	36.2	19.9	14.4	7.8	2.9	18.8	[77]
Castellón de la Plana/Spain	57.0	15.0	10.0	7.0	4.0	7.0	[78]
London, Ontario/Canada	30.0	32.0	10.0	6.0	3.0	19.0	[79]

**Table 8:** Chi-square test result for the relationship between dealing with recyclable materials and residential districts.

	Value	df	Asymptotic significance (2-sided)
Pearson chi-square	10.826	9	0.288
Likelihood ratio	11.097	9	0.269
Linear-by-linear association	0.373	1	0.542
Number of valid cases	300		

**Table 9:** Per capita waste generation model.

Model	Unstandardized coefficients		Standardized coefficients	*t*	Sig.
	*B*	Std. error	Beta		
Constant	0.608	0.034		18.094	0.000^*∗∗∗*^
Gender	0.022	0.007	0.118	3.027	0.003^*∗∗∗*^
Education level	−0.035	0.005	−0.357	−6.826	0.000^*∗∗∗*^
Monthly income	0.040	0.005	0.356	7.692	0.000^*∗∗∗*^
Household size	0.009	0.006	0.070	1.519	0.013^*∗∗*^
Age	0.012	0.006	0.116	2.172	0.031^*∗∗*^

^*∗*^ *P* < 0.1, ^*∗∗*^*P* < 0.05, and ^*∗∗∗*^*P* < 0.01, significant level; adjusted *R*^2^ value = 0.557; *F* value = 76.262, *P* < 0.01.

**Table 10:** Daily organic waste generation model.

Model	Unstandardized coefficients		Standardized coefficients	*t*	Sig.
	*B*	Std. error	Beta		
Constant	0.526	0.255		2.064	0.040^*∗∗*^
Gender	0.058	0.056	0.024	1.034	0.302
Education level	−0.295	0.039	−0.241	−7.639	0.000^*∗∗∗*^
Monthly income	0.098	0.040	0.069	2.477	0.014^*∗∗*^
Household size	1.193	0.047	0.709	25.562	0.000^*∗∗∗*^
Age	0.122	0.043	0.092	2.838	0.005^*∗∗∗*^

^*∗∗*^ *P* < 0.1, ^*∗∗*^*P* < 0.05, and ^*∗∗∗*^*P* < 0.01, significant level; adjusted *R*^2^ value = 0.839; *F* value = 313.338, *P* < 0.01.

**Table 11:** Daily packaging waste generation model.

Model	Unstandardized coefficients		Standardized coefficients	*t*	Sig.
	*B*	Std. error	Beta		
Constant	−0.125	0.116		−1.086	0.279
Gender	0.112	0.025	0.139	4.403	0.000^*∗∗∗*^
Education level	−.081	0.018	−.195	−4.595	0.000^*∗∗∗*^
Monthly income	0.090	0.018	0.188	5.016	0.000^*∗∗∗*^
Household size	0.331	0.021	0.582	15.624	0.000^*∗∗∗*^
Age	0.031	0.020	0.070	1.608	0.100^*∗∗*^

^*∗∗*^ *P* < 0.01, ^*∗∗*^*P* < 0.05, and ^*∗∗∗*^*P* < 0.01, significant level; adjusted *R*^2^ value = 0.709; *F* value = 146.983, *P* < 0.01.

## Data Availability

The datasets, used and/or analyzed during this study, are available from the corresponding author upon a justified request.

## Supplementary Materials

Supplementary Materials The supplementary materials file includes data obtained from the survey and waste characteristics study and later were inserted in SPSS to perform some statistical tests. Data collected include the demographic characteristics of the respondent households (household size, monthly income, gender, and education level) as well as their answers on questions about waste management practices applied at the household level. Besides, the file includes raw data obtained from field measurements (per capita waste generation and waste composition of each household) in order to determine the characteristics of household waste in Homs city, Syria.
