# Supplementary material for: Determinants of Household Solid Waste Generation and Composition in Homs City, Syria
Source: J Environ Public Health. 2020 Nov 26;2020:7460356. doi: 10.1155/2020/7460356 (PMC7714613; doi:10.1155/2020/7460356)
Supplement: Supplementary Materials — The supplementary materials file includes data obtained from the survey and waste characteristics study and later were inserted in SPSS to perform some statistical tests. Data collected include the demographic characteristics of the respondent households (household size, monthly income, gender, and education level) as well as their answers on questions about waste management practices applied at the household level. Besides, the file includes raw data obtained from field measurements (per capita waste generation and waste composition of each household) in order to determine the characteristics of household waste in Homs city, Syria. [file 7460356.f1.pdf]

id	area	size	gender	educ	Income	Waste generation per capita
1	Zone1	2	f	College / Institute	50.001-100.000	0.5420
2	Zone1	2	m	College / Institute	50.001-100.000	0.5475
3	Zone1	2	f	College / Institute	50.001-100.000	0.6028
4	Zone1	2	f	Postgraduate	50.001-100.000	0.5150
5	Zone1	3	m	College / Institute	50.001-100.000	0.6024
6	Zone1	3	f	College / Institute	50.001-100.000	0.6036
7	Zone1	3	m	High school	50.001-100.000	0.6012
8	Zone1	3	f	College / Institute	50.001-100.000	0.5964
9	Zone1	3	f	College / Institute	50.001-100.000	0.5917
10	Zone1	3	f	College / Institute	50.001-100.000	0.5952
11	Zone1	3	f	High school	Less than 50.000	0.6095
12	Zone1	4	f	High school	100.001-150.000	0.6554
13	Zone1	4	m	High school	50.001-100.000	0.6491
14	Zone1	4	f	High school	50.001-100.000	0.6473
15	Zone1	4	f	High school	50.001-100.000	0.6431
16	Zone1	4	m	High school	50.001-100.000	0.6411
17	Zone1	4	f	High school	100.001-150.000	0.6500
18	Zone1	5	f	High school	100.001-150.000	0.6912
19	Zone1	5	m	Elementary school	150.001-200.000	0.7050
20	Zone1	5	f	Elementary school	150.001-200.000	0.7017
21	Zone1	5	f	High school	100.001-150.000	0.6950
22	Zone1	5	m	Elementary school	150.001-200.000	0.7034
23	Zone1	5	f	High school	100.001-150.000	0.6971
24	Zone1	5	f	Elementary school	150.001-200.000	0.6991
25	Zone1	5	f	Junior high or middle school	150.001-200.000	0.7093
26	Zone1	5	m	Junior high or middle school	More than 200.000	0.6983
27	Zone1	5	f	Elementary school	150.001-200.000	0.7036
28	Zone1	5	m	Junior high or middle school	150.001-200.000	0.7011
29	Zone1	5	f	Junior high or middle school	100.001-150.000	0.6936
30	Zone1	6	m	Junior high or middle school	100.001-150.000	0.6780
31	Zone1	6	m	High school	100.001-150.000	0.6606
32	Zone1	6	f	High school	100.001-150.000	0.6794
33	Zone1	6	m	High school	100.001-150.000	0.6692
34	Zone1	6	f	Junior high or middle school	100.001-150.000	0.6926
35	Zone1	6	m	Junior high or middle school	100.001-150.000	0.7039
36	Zone1	6	f	High school	Less than 50.000	0.6501
37	Zone1	6	m	High school	Less than 50.000	0.6546
38	Zone1	6	f	Junior high or middle school	100.001-150.000	0.6870
39	Zone1	6	m	Junior high or middle school	100.001-150.000	0.6982
40	Zone1	6	f	High school	100.001-150.000	0.6750
41	Zone1	6	f	Junior high or middle school	100.001-150.000	0.6908
42	Zone1	6	m	High school	Less than 50.000	0.6492
43	Zone1	6	f	Junior high or middle school	100.001-150.000	0.6802
44	Zone1	6	m	Junior high or middle school	100.001-150.000	0.6860
45	Zone1	6	m	High school	Less than 50.000	0.6268
46	Zone1	7	f	College / Institute	50.001-100.000	0.5444
47	Zone1	7	f	College / Institute	50.001-100.000	0.5668

48	Zone1	7	m	College / Institute	50.001-100.000	0.5337
49	Zone1	7	f	College / Institute	50.001-100.000	0.5699
50	Zone1	7	f	College / Institute	50.001-100.000	0.5474
51	Zone1	7	m	College / Institute	50.001-100.000	0.5582
52	Zone1	7	f	College / Institute	50.001-100.000	0.5495
53	Zone1	7	f	College / Institute	50.001-100.000	0.5663
54	Zone1	7	m	College / Institute	50.001-100.000	0.5709
55	Zone1	7	f	College / Institute	50.001-100.000	0.5673
56	Zone1	7	m	High school	50.001-100.000	0.5969
57	Zone1	8	m	College / Institute	50.001-100.000	0.5563
58	Zone1	8	f	College / Institute	50.001-100.000	0.5471
59	Zone1	8	f	College / Institute	50.001-100.000	0.5179
60	Zone1	8	m	College / Institute	50.001-100.000	0.5571
61	Zone1	8	m	College / Institute	50.001-100.000	0.5642
62	Zone1	8	m	College / Institute	50.001-100.000	0.5556
63	Zone1	9	m	College / Institute	50.001-100.000	0.5306
64	Zone1	9	m	College / Institute	50.001-100.000	0.5635
65	Zone1	9	f	College / Institute	50.001-100.000	0.5779
66	Zone1	9	f	College / Institute	50.001-100.000	0.5386
67	Zone1	9	f	College / Institute	50.001-100.000	0.5290
68	Zone1	9	f	High school	50.001-100.000	0.5925
69	Zone1	9	m	College / Institute	50.001-100.000	0.5537
70	Zone1	10	m	College / Institute	50.001-100.000	0.5356
71	Zone1	10	m	College / Institute	50.001-100.000	0.5654
72	Zone1	10	f	Postgraduate	50.001-100.000	0.5268
73	Zone1	10	f	College / Institute	50.001-100.000	0.5737
74	Zone1	10	f	Postgraduate	50.001-100.000	0.5179
75	Zone1	10	f	Postgraduate	50.001-100.000	0.3276
76	Zone2	2	f	Postgraduate	50.001-100.000	0.5411
77	Zone2	2	f	Postgraduate	50.001-100.000	0.5391
78	Zone2	2	f	Postgraduate	50.001-100.000	0.5675
79	Zone2	2	f	Postgraduate	50.001-100.000	0.5432
80	Zone2	2	f	College / Institute	50.001-100.000	0.5609
81	Zone2	3	f	College / Institute	50.001-100.000	0.6003
82	Zone2	3	f	College / Institute	50.001-100.000	0.6013
83	Zone2	3	f	College / Institute	50.001-100.000	0.6030
84	Zone2	3	f	Postgraduate	50.001-100.000	0.5806
85	Zone2	3	m	College / Institute	50.001-100.000	0.5825
86	Zone2	3	m	Postgraduate	50.001-100.000	0.5892
87	Zone2	4	f	College / Institute	50.001-100.000	0.6891
88	Zone2	4	m	College / Institute	50.001-100.000	0.6098
89	Zone2	4	m	College / Institute	50.001-100.000	0.6893
90	Zone2	4	f	College / Institute	50.001-100.000	0.6029
91	Zone2	4	f	College / Institute	50.001-100.000	0.6882
92	Zone2	4	f	College / Institute	50.001-100.000	0.6733
93	Zone2	4	f	College / Institute	50.001-100.000	0.6729
94	Zone2	5	f	College / Institute	100.001-150.000	0.7115
95	Zone2	5	f	College / Institute	150.001-200.000	0.7043

96	Zone2	5	m	College / Institute	100.001-150.000	0.7072
97	Zone2	5	f	College / Institute	More than 200.000	0.7077
98	Zone2	5	m	High school	100.001-150.000	0.7185
99	Zone2	5	f	College / Institute	150.001-200.000	0.7086
100	Zone2	5	m	College / Institute	100.001-150.000	0.7071
101	Zone2	5	m	High school	150.001-200.000	0.7102
102	Zone2	5	f	College / Institute	100.001-150.000	0.7024
103	Zone2	5	m	College / Institute	150.001-200.000	0.7086
104	Zone2	5	f	High school	100.001-150.000	0.7245
105	Zone2	6	m	College / Institute	50.001-100.000	0.6912
106	Zone2	6	f	College / Institute	50.001-100.000	0.6923
107	Zone2	6	f	High school	100.001-150.000	0.7120
108	Zone2	6	f	High school	100.001-150.000	0.7115
109	Zone2	6	m	High school	150.001-200.000	0.7097
110	Zone2	6	f	College / Institute	50.001-100.000	0.5872
111	Zone2	6	m	High school	150.001-200.000	0.7108
112	Zone2	6	f	High school	100.001-150.000	0.7116
113	Zone2	6	m	College / Institute	50.001-100.000	0.6925
114	Zone2	6	f	College / Institute	50.001-100.000	0.5922
115	Zone2	6	f	College / Institute	50.001-100.000	0.5881
116	Zone2	6	m	College / Institute	50.001-100.000	0.5845
117	Zone2	6	f	College / Institute	50.001-100.000	0.5990
118	Zone2	7	f	College / Institute	Less than 50.000	0.6834
119	Zone2	7	f	College / Institute	Less than 50.000	0.6850
120	Zone2	7	f	High school	50.001-100.000	0.6970
121	Zone2	7	f	College / Institute	50.001-100.000	0.6989
122	Zone2	7	f	High school	50.001-100.000	0.7023
123	Zone2	7	f	High school	150.001-200.000	0.7030
124	Zone2	7	f	High school	100.001-150.000	0.7066
125	Zone2	7	m	High school	150.001-200.000	0.7097
126	Zone2	7	f	High school	100.001-150.000	0.7024
127	Zone2	7	m	College / Institute	50.001-100.000	0.6993
128	Zone2	7	f	High school	50.001-100.000	0.7028
129	Zone2	7	m	College / Institute	50.001-100.000	0.6983
130	Zone2	7	m	High school	150.001-200.000	0.7034
131	Zone2	8	m	Junior high or middle school	100.001-150.000	0.7110
132	Zone2	8	m	High school	100.001-150.000	0.7099
133	Zone2	8	m	Junior high or middle school	100.001-150.000	0.7100
134	Zone2	8	f	Junior high or middle school	100.001-150.000	0.7104
135	Zone2	8	f	High school	100.001-150.000	0.7094
136	Zone2	8	m	High school	100.001-150.000	0.7097
137	Zone2	9	m	Elementary school	100.001-150.000	0.7204
138	Zone2	9	m	Elementary school	100.001-150.000	0.7201
139	Zone2	9	f	Junior high or middle school	100.001-150.000	0.7203
140	Zone2	9	f	Junior high or middle school	100.001-150.000	0.7209
141	Zone2	9	f	Junior high or middle school	100.001-150.000	0.7193
142	Zone2	9	f	Junior high or middle school	100.001-150.000	0.7195
143	Zone2	9	f	Elementary school	100.001-150.000	0.7203

144	Zone2	10	f	High school	50.001-100.000	0.7018
145	Zone2	10	m	High school	100.001-150.000	0.7081
146	Zone2	10	m	High school	50.001-100.000	0.7009
147	Zone2	10	m	College / Institute	50.001-100.000	0.6880
148	Zone2	10	f	High school	50.001-100.000	0.7028
149	Zone2	10	m	Junior high or middle school	100.001-150.000	0.7126
150	Zone2	10	m	High school	50.001-100.000	0.7034
151	Zone3	2	m	College / Institute	50.001-100.000	0.5366
152	Zone3	2	m	Postgraduate	50.001-100.000	0.5293
153	Zone3	2	m	College / Institute	50.001-100.000	0.5435
154	Zone3	2	m	College / Institute	50.001-100.000	0.5322
155	Zone3	3	m	Postgraduate	50.001-100.000	0.3933
156	Zone3	3	m	College / Institute	50.001-100.000	0.5401
157	Zone3	3	m	Postgraduate	50.001-100.000	0.4887
158	Zone3	3	m	College / Institute	50.001-100.000	0.5853
159	Zone3	3	m	College / Institute	50.001-100.000	0.5853
160	Zone3	3	m	College / Institute	50.001-100.000	0.5861
161	Zone3	3	m	College / Institute	50.001-100.000	0.5895
162	Zone3	4	m	College / Institute	50.001-100.000	0.6050
163	Zone3	4	m	College / Institute	50.001-100.000	0.6017
164	Zone3	4	m	College / Institute	50.001-100.000	0.5931
165	Zone3	4	m	College / Institute	50.001-100.000	0.6094
166	Zone3	4	m	College / Institute	50.001-100.000	0.6015
167	Zone3	4	m	College / Institute	50.001-100.000	0.6041
168	Zone3	4	m	College / Institute	50.001-100.000	0.6010
169	Zone3	4	m	College / Institute	50.001-100.000	0.6026
170	Zone3	5	f	College / Institute	50.001-100.000	0.5987
171	Zone3	5	m	College / Institute	50.001-100.000	0.6100
172	Zone3	5	m	College / Institute	150.001-200.000	0.7106
173	Zone3	5	m	College / Institute	50.001-100.000	0.7001
174	Zone3	5	m	College / Institute	50.001-100.000	0.6933
175	Zone3	5	f	College / Institute	50.001-100.000	0.7057
176	Zone3	5	f	College / Institute	50.001-100.000	0.7000
177	Zone3	5	f	College / Institute	50.001-100.000	0.6958
178	Zone3	5	m	College / Institute	50.001-100.000	0.7035
179	Zone3	5	f	College / Institute	50.001-100.000	0.7014
180	Zone3	5	m	College / Institute	Less than 50.000	0.6904
181	Zone3	6	f	High school	100.001-150.000	0.7827
182	Zone3	6	m	High school	100.001-150.000	0.8191
183	Zone3	6	m	High school	100.001-150.000	0.8171
184	Zone3	6	f	Junior high or middle school	150.001-200.000	0.8203
185	Zone3	6	f	Junior high or middle school	100.001-150.000	0.8212
186	Zone3	6	m	Junior high or middle school	100.001-150.000	0.8255
187	Zone3	6	f	High school	150.001-200.000	0.7302
188	Zone3	6	m	Junior high or middle school	100.001-150.000	0.8202
189	Zone3	6	f	College / Institute	100.001-150.000	0.7238
190	Zone3	6	m	High school	100.001-150.000	0.8206
191	Zone3	6	f	High school	150.001-200.000	0.8156

192	Zone3	6	m	Junior high or middle school	100.001-150.000	0.8217
193	Zone3	6	m	High school	100.001-150.000	0.7302
194	Zone3	6	m	High school	100.001-150.000	0.7187
195	Zone3	6	m	High school	150.001-200.000	0.7219
196	Zone3	6	f	High school	100.001-150.000	0.7237
197	Zone3	7	m	High school	100.001-150.000	0.8120
198	Zone3	7	m	High school	100.001-150.000	0.8118
199	Zone3	7	m	High school	100.001-150.000	0.8101
200	Zone3	7	m	High school	150.001-200.000	0.8113
201	Zone3	7	m	High school	More than 200.000	0.8108
202	Zone3	7	m	High school	150.001-200.000	0.8111
203	Zone3	7	m	College / Institute	150.001-200.000	0.7618
204	Zone3	7	m	College / Institute	More than 200.000	0.7120
205	Zone3	7	m	High school	150.001-200.000	0.8113
206	Zone3	7	m	High school	150.001-200.000	0.8115
207	Zone3	7	m	High school	150.001-200.000	0.8119
208	Zone3	8	m	High school	100.001-150.000	0.7315
209	Zone3	8	m	High school	100.001-150.000	0.7105
210	Zone3	8	m	Elementary school	100.001-150.000	0.8349
211	Zone3	8	m	Elementary school	100.001-150.000	0.8350
212	Zone3	8	m	Elementary school	100.001-150.000	0.8354
213	Zone3	8	m	Junior high or middle school	100.001-150.000	0.8344
214	Zone3	8	m	High school	100.001-150.000	0.8092
215	Zone3	8	f	High school	100.001-150.000	0.8095
216	Zone3	8	m	High school	100.001-150.000	0.8104
217	Zone3	9	m	Junior high or middle school	100.001-150.000	0.8311
218	Zone3	9	m	Elementary school	100.001-150.000	0.8304
219	Zone3	9	m	Elementary school	100.001-150.000	0.8306
220	Zone3	9	m	Elementary school	100.001-150.000	0.8314
221	Zone3	9	m	High school	100.001-150.000	0.7877
222	Zone3	10	m	High school	100.001-150.000	0.8148
223	Zone3	10	f	High school	100.001-150.000	0.8099
224	Zone3	10	m	High school	100.001-150.000	0.8133
225	Zone3	10	m	High school	100.001-150.000	0.8034
226	Zone4	2	f	College / Institute	50.001-100.000	0.5880
227	Zone4	2	m	Postgraduate	50.001-100.000	0.5665
228	Zone4	2	m	Postgraduate	50.001-100.000	0.5410
229	Zone4	2	m	College / Institute	50.001-100.000	0.5795
230	Zone4	2	m	College / Institute	50.001-100.000	0.5768
231	Zone4	3	m	College / Institute	50.001-100.000	0.6885
232	Zone4	3	m	College / Institute	50.001-100.000	0.6895
233	Zone4	3	m	College / Institute	50.001-100.000	0.6915
234	Zone4	3	m	College / Institute	50.001-100.000	0.6869
235	Zone4	3	m	High school	50.001-100.000	0.7028
236	Zone4	3	f	College / Institute	50.001-100.000	0.6032
237	Zone4	4	m	College / Institute	50.001-100.000	0.6737
238	Zone4	4	f	College / Institute	50.001-100.000	0.6890
239	Zone4	4	m	College / Institute	100.001-150.000	0.7140

240	Zone4	4	m	College / Institute	50.001-100.000	0.6896
241	Zone4	4	m	College / Institute	50.001-100.000	0.6921
242	Zone4	4	f	College / Institute	Less than 50.000	0.6722
243	Zone4	4	m	High school	100.001-150.000	0.7151
244	Zone4	4	m	College / Institute	50.001-100.000	0.6756
245	Zone4	4	m	College / Institute	50.001-100.000	0.6888
246	Zone4	5	m	College / Institute	50.001-100.000	0.6938
247	Zone4	5	f	High school	50.001-100.000	0.7100
248	Zone4	5	f	Elementary school	100.001-150.000	0.8310
249	Zone4	5	m	College / Institute	50.001-100.000	0.7029
250	Zone4	5	f	College / Institute	50.001-100.000	0.7106
251	Zone4	5	f	College / Institute	50.001-100.000	0.7043
252	Zone4	5	m	College / Institute	50.001-100.000	0.7058
253	Zone4	5	f	High school	100.001-150.000	0.7188
254	Zone4	5	m	College / Institute	50.001-100.000	0.7047
255	Zone4	6	m	High school	100.001-150.000	0.7827
256	Zone4	6	m	High school	100.001-150.000	0.8193
257	Zone4	6	m	High school	100.001-150.000	0.8173
258	Zone4	6	m	High school	100.001-150.000	0.8204
259	Zone4	6	f	High school	100.001-150.000	0.8212
260	Zone4	6	m	Junior high or middle school	100.001-150.000	0.8254
261	Zone4	6	m	Junior high or middle school	100.001-150.000	0.8284
262	Zone4	6	m	Junior high or middle school	150.001-200.000	0.8202
263	Zone4	6	f	Junior high or middle school	150.001-200.000	0.8168
264	Zone4	6	m	Junior high or middle school	100.001-150.000	0.8205
265	Zone4	6	m	High school	100.001-150.000	0.8154
266	Zone4	6	f	Junior high or middle school	150.001-200.000	0.8212
267	Zone4	6	f	College / Institute	150.001-200.000	0.7301
268	Zone4	6	m	College / Institute	50.001-100.000	0.7186
269	Zone4	6	m	College / Institute	150.001-200.000	0.7219
270	Zone4	6	m	College / Institute	100.001-150.000	0.7238
271	Zone4	6	m	High school	100.001-150.000	0.7281
272	Zone4	7	m	High school	100.001-150.000	0.7709
273	Zone4	7	m	College / Institute	150.001-200.000	0.7712
274	Zone4	7	m	High school	150.001-200.000	0.7648
275	Zone4	7	m	College / Institute	100.001-150.000	0.7799
276	Zone4	7	m	College / Institute	100.001-150.000	0.7542
277	Zone4	7	f	College / Institute	100.001-150.000	0.7584
278	Zone4	7	m	College / Institute	150.001-200.000	0.7516
279	Zone4	7	m	College / Institute	50.001-100.000	0.7118
280	Zone4	7	f	High school	150.001-200.000	0.7665
281	Zone4	7	m	High school	150.001-200.000	0.7524
282	Zone4	7	m	High school	100.001-150.000	0.7830
283	Zone4	7	m	High school	100.001-150.000	0.7535
284	Zone4	7	m	High school	150.001-200.000	0.7627
285	Zone4	8	m	High school	100.001-150.000	0.7584
286	Zone4	8	m	High school	100.001-150.000	0.7619
287	Zone4	8	m	High school	100.001-150.000	0.7681

288	Zone4	8	m	High school	100.001-150.000	0.7759
289	Zone4	8	m	High school	100.001-150.000	0.7981
290	Zone4	8	m	High school	100.001-150.000	0.7831
291	Zone4	8	m	High school	150.001-200.000	0.8094
292	Zone4	9	m	High school	100.001-150.000	0.7200
293	Zone4	9	m	High school	150.001-200.000	0.8090
294	Zone4	9	m	Elementary school	100.001-150.000	0.8177
295	Zone4	9	m	Elementary school	100.001-150.000	0.8148
296	Zone4	9	m	High school	100.001-150.000	0.7893
297	Zone4	10	f	High school	150.001-200.000	0.8095
298	Zone4	10	m	Elementary school	More than 200.000	0.8748
299	Zone4	10	f	Elementary school	150.001-200.000	0.8155
300	Zone4	10	m	Junior high or middle school	More than 200.000	0.8108

Storage	separate	Materials separated	Responsibilities of separation
Plastic bag	Yes	Paper, Plastic, Glass, Textile, and Metal	Father
Plastic bag	No	0.00	0.00
Plastic bag	Yes	Plastic and Glass	Children
Plastic bag	Yes	Plastic and Glass	Mother or housemaid
Plastic bag	Yes	Battery and electronic waste	Mother or housemaid
Plastic bag	Yes	Paper, Plastic, Glass, Textile, and Metal	Mother or housemaid
Plastic bag	Yes	Plastic and Glass	Mother or housemaid
Plastic bag	Yes	Battery and electronic waste	Father
Plastic bag	No	0.00	0.00
Plastic bag	Yes	Paper, Plastic, Glass, Textile, and Metal	Children
box	Yes	Plastic and Glass	Mother or housemaid
Plastic bag	Yes	Paper, Plastic, Glass, Textile, and Metal	Father
Plastic bag	No	0.00	0.00
Plastic bag	Yes	Plastic and Glass	Mother or housemaid
Plastic bag	No	0.00	0.00
Plastic bag	No	0.00	0.00
Plastic bag	No	0.00	0.00
Plastic bag	Yes	Paper, Plastic, Glass, Textile, and Metal	Father
Basket	Yes	Plastic and Glass	Mother or housemaid
Mixed methods	Yes	Plastic and Glass	Mother or housemaid
box	No	0.00	0.00
Plastic bag	No	0.00	0.00
Plastic bag	Yes	Plastic and Glass	Mother or housemaid
Plastic bag	Yes	Paper, Plastic, Glass, Textile, and Metal	Father
Plastic bag	Yes	Plastic and Glass	Children
Plastic bag	Yes	Paper, Plastic, Glass, Textile, and Metal	Mother or housemaid
Plastic bag	Yes	Plastic and Glass	Father
Basket	Yes	Paper, Plastic, Glass, Textile, and Metal	Mother or housemaid
Plastic bag	Yes	Plastic and Glass	All family member
Plastic bag	Yes	Paper, Plastic, Glass, Textile, and Metal	Mother or housemaid
Basket	Yes	Plastic and Glass	Mother or housemaid
Open container	No	0.00	0.00
Mixed methods	No	0.00	0.00
Plastic bag	Yes	Plastic and Glass	Mother or housemaid
Plastic bag	No	0.00	0.00
Plastic bag	Yes	Paper, Plastic, Glass, Textile, and Metal	All family member
Mixed methods	Yes	Plastic and Glass	Mother or housemaid
Open container	No	0.00	0.00
Basket	No	0.00	0.00
Plastic bag	Yes	Paper, Plastic, Glass, Textile, and Metal	Mother or housemaid
Plastic bag	No	0.00	0.00
Plastic bag	No	0.00	0.00
box	Yes	Paper, Plastic, Glass, Textile, and Metal	All family member
Open container	Yes	Plastic and Glass	Mother or housemaid
Basket	Yes	Plastic and Glass	Mother or housemaid
Open container	Yes	Paper, Plastic, Glass, Textile, and Metal	All family member
Plastic bag	Yes	Plastic and Glass	Mother or housemaid

Mixed methods	Yes	Paper, Plastic, Glass, Textile, and Metal	Father
Open container	No	0.00	0.00
Basket	Yes	Plastic and Glass	Mother or housemaid
Mixed methods	No	0.00	0.00
box	Yes	Paper, Plastic, Glass, Textile, and Metal	Children
Mixed methods	Yes	Plastic and Glass	Mother or housemaid
box	Yes	Plastic and Glass	Mother or housemaid
Mixed methods	Yes	Paper, Plastic, Glass, Textile, and Metal	Father
Plastic bag	Yes	Plastic and Glass	Mother or housemaid
Open container	No	0.00	0.00
Plastic bag	No	0.00	0.00
Mixed methods	No	0.00	0.00
Plastic bag	Yes	Battery and electronic waste	Father
Plastic bag	Yes	Plastic and Glass	All family member
Mixed methods	Yes	Paper, Plastic, Glass, Textile, and Metal	Children
Plastic bag	Yes	Plastic and Glass	Mother or housemaid
Plastic bag	Yes	Plastic and Glass	Mother or housemaid
Open container	Yes	Paper, Plastic, Glass, Textile, and Metal	Father
Mixed methods	Yes	Plastic and Glass	Mother or housemaid
Open container	Yes	Paper, Plastic, Glass, Textile, and Metal	Children
Plastic bag	Yes	Plastic and Glass	Father
Plastic bag	No	0.00	0.00
Plastic bag	No	0.00	0.00
Open container	Yes	Paper, Plastic, Glass, Textile, and Metal	Father
Plastic bag	Yes	Plastic and Glass	Children
Plastic bag	No	0.00	0.00
Plastic bag	No	0.00	0.00
Plastic bag	Yes	Plastic and Glass	Mother or housemaid
Plastic bag	Yes	Paper, Plastic, Glass, Textile, and Metal	Father
box	Yes	Plastic and Glass	Mother or housemaid
Plastic bag	Yes	Plastic and Glass	Mother or housemaid
Plastic bag	No	0.00	0.00
Plastic bag	Yes	Plastic and Glass	Mother or housemaid
Plastic bag	No	0.00	0.00
Plastic bag	No	0.00	0.00
Plastic bag	No	0.00	0.00
Plastic bag	Yes	Plastic and Glass	Children
Basket	Yes	Plastic and Glass	Mother or housemaid
Mixed methods	Yes	Paper, Plastic, Glass, Textile, and Metal	Mother or housemaid
box	Yes	Plastic and Glass	All family member
Plastic bag	Yes	Battery and electronic waste	Father
Plastic bag	Yes	Plastic and Glass	Father
Plastic bag	Yes	Plastic and Glass	Mother or housemaid
Plastic bag	Yes	Paper, Plastic, Glass, Textile, and Metal	All family member
Plastic bag	Yes	Plastic and Glass	Children
Plastic bag	No	0.00	0.00
Basket	Yes	Paper, Plastic, Glass, Textile, and Metal	Mother or housemaid
Plastic bag	Yes	Plastic and Glass	Mother or housemaid

Plastic bag	Yes	Paper, Plastic, Glass, Textile, and Metal	Father	
Basket	No	0.00		0.00
Open container	Yes	Plastic and Glass	Children	
Mixed methods	No	0.00		0.00
Plastic bag	No	0.00		0.00
Plastic bag	No	0.00		0.00
Plastic bag	Yes	Paper, Plastic, Glass, Textile, and Metal	Mother or housemaid	
Basket	Yes	Plastic and Glass	Father	
Mixed methods	Yes	Plastic and Glass	Mother or housemaid	
Plastic bag	No	0.00		0.00
Mixed methods	No	0.00		0.00
box	Yes	Paper, Plastic, Glass, Textile, and Metal	All family member	
Mixed methods	Yes	Battery and electronic waste	Father	
Plastic bag	Yes	Plastic and Glass	Children	
Mixed methods	Yes	Plastic and Glass	Mother or housemaid	
Plastic bag	Yes	Paper, Plastic, Glass, Textile, and Metal	Mother or housemaid	
Mixed methods	Yes	Plastic and Glass	Mother or housemaid	
Plastic bag	Yes	Plastic and Glass	Mother or housemaid	
Plastic bag	No	0.00		0.00
Mixed methods	Yes	Plastic and Glass	Mother or housemaid	
Plastic bag	No	0.00		0.00
Plastic bag	Yes	Plastic and Glass	Father	
Plastic bag	Yes	Battery and electronic waste	Father	
Mixed methods	Yes	Plastic and Glass	All family member	
Plastic bag	Yes	Paper, Plastic, Glass, Textile, and Metal	Mother or housemaid	
Plastic bag	Yes	Plastic and Glass	Children	
Plastic bag	No	0.00		0.00
Plastic bag	No	0.00		0.00
Open container	No	0.00		0.00
Plastic bag	Yes	Paper, Plastic, Glass, Textile, and Metal	Father	
Plastic bag	Yes	Plastic and Glass	Mother or housemaid	
Plastic bag	Yes	Paper, Plastic, Glass, Textile, and Metal	Mother or housemaid	
Plastic bag	Yes	Plastic and Glass	All family member	
Plastic bag	Yes	Plastic and Glass	Mother or housemaid	
Plastic bag	Yes	Paper, Plastic, Glass, Textile, and Metal	Mother or housemaid	
Mixed methods	Yes	Plastic and Glass	Father	
Open container	Yes	Plastic and Glass	Mother or housemaid	
Basket	Yes	Paper, Plastic, Glass, Textile, and Metal	Father	
Plastic bag	No	0.00		0.00
Plastic bag	No	0.00		0.00
Plastic bag	Yes	Plastic and Glass	Father	
Plastic bag	Yes	Plastic and Glass	Mother or housemaid	
Open container	Yes	Plastic and Glass	Mother or housemaid	
Basket	No	0.00		0.00
Open container	Yes	Plastic and Glass	Father	
Plastic bag	No	0.00		0.00
Mixed methods	Yes	Plastic and Glass	Mother or housemaid	
Open container	Yes	Plastic and Glass	Mother or housemaid	

Basket	Yes	Plastic and Glass	Mother or housemaid
Plastic bag	No	0.00	0.00
Plastic bag	No	0.00	0.00
Plastic bag	Yes	Plastic and Glass	Mother or housemaid
Plastic bag	Yes	Plastic and Glass	Mother or housemaid
Plastic bag	Yes	Plastic and Glass	Mother or housemaid
Open container	Yes	Plastic and Glass	Father
Plastic bag	Yes	Plastic and Glass	All family member
Mixed methods	No	0.00	0.00
box	Yes	Plastic and Glass	Mother or housemaid
Mixed methods	No	0.00	0.00
Plastic bag	No	0.00	0.00
Mixed methods	No	0.00	0.00
Plastic bag	Yes	Plastic and Glass	Children
Open container	Yes	Paper, Plastic, Glass, Textile, and Metal	Mother or housemaid
Plastic bag	Yes	Battery and electronic waste	Mother or housemaid
Mixed methods	Yes	Plastic and Glass	Mother or housemaid
Plastic bag	Yes	Paper, Plastic, Glass, Textile, and Metal	Mother or housemaid
Plastic bag	Yes	Plastic and Glass	All family member
Mixed methods	Yes	Paper, Plastic, Glass, Textile, and Metal	Mother or housemaid
Plastic bag	No	0.00	0.00
Plastic bag	Yes	Plastic and Glass	Mother or housemaid
Open container	Yes	Plastic and Glass	Mother or housemaid
Mixed methods	Yes	Battery and electronic waste	Father
Open container	Yes	Plastic and Glass	Mother or housemaid
Plastic bag	Yes	Paper, Plastic, Glass, Textile, and Metal	Mother or housemaid
Plastic bag	Yes	Plastic and Glass	Mother or housemaid
Plastic bag	No	0.00	0.00
Open container	Yes	Paper, Plastic, Glass, Textile, and Metal	Mother or housemaid
Plastic bag	Yes	Plastic and Glass	Mother or housemaid
Plastic bag	Yes	Paper, Plastic, Glass, Textile, and Metal	Mother or housemaid
Plastic bag	No	0.00	0.00
Plastic bag	Yes	Plastic and Glass	Children
Plastic bag	No	0.00	0.00
Plastic bag	No	0.00	0.00
Plastic bag	No	0.00	0.00
Plastic bag	Yes	Paper, Plastic, Glass, Textile, and Metal	Mother or housemaid
Plastic bag	Yes	Plastic and Glass	Mother or housemaid
Basket	Yes	Paper, Plastic, Glass, Textile, and Metal	Mother or housemaid
Plastic bag	No	0.00	0.00
Mixed methods	No	0.00	0.00
box	Yes	Plastic and Glass	Children
Plastic bag	No	0.00	0.00
Plastic bag	Yes	Plastic and Glass	Mother or housemaid
Mixed methods	No	0.00	0.00
Open container	No	0.00	0.00
Plastic bag	Yes	Paper, Plastic, Glass, Textile, and Metal	Mother or housemaid
Plastic bag	Yes	Plastic and Glass	Mother or housemaid

Plastic bag	Yes	Paper, Plastic, Glass, Textile, and Metal	Mother or housemaid	
Plastic bag	No	0.00		0.00
Plastic bag	No	0.00		0.00
Mixed methods	Yes	Plastic and Glass	Mother or housemaid	
Basket	No	0.00		0.00
Open container	Yes	Plastic and Glass	Mother or housemaid	
Plastic bag	Yes	Paper, Plastic, Glass, Textile, and Metal	Mother or housemaid	
Mixed methods	Yes	Plastic and Glass	Mother or housemaid	
Plastic bag	No	0.00		0.00
Plastic bag	Yes	Plastic and Glass	Children	
Plastic bag	No	0.00		0.00
Plastic bag	No	0.00		0.00
Plastic bag	No	0.00		0.00
Plastic bag	Yes	Battery and electronic waste	Father	
Plastic bag	Yes	Plastic and Glass	Mother or housemaid	
Plastic bag	Yes	Paper, Plastic, Glass, Textile, and Metal	Mother or housemaid	
Plastic bag	Yes	Plastic and Glass	Mother or housemaid	
Basket	Yes	Plastic and Glass	Mother or housemaid	
Mixed methods	Yes	Plastic and Glass	Mother or housemaid	
Plastic bag	No	0.00		0.00
Plastic bag	Yes	Plastic and Glass	Mother or housemaid	
Plastic bag	Yes	Plastic and Glass	Mother or housemaid	
Plastic bag	No	0.00		0.00
Plastic bag	No	0.00		0.00
Plastic bag	Yes	Paper, Plastic, Glass, Textile, and Metal	Father	
Plastic bag	Yes	Plastic and Glass	Mother or housemaid	
Basket	No	0.00		0.00
Plastic bag	No	0.00		0.00
Plastic bag	Yes	Plastic and Glass	Mother or housemaid	
Basket	No	0.00		0.00
Mixed methods	Yes	Plastic and Glass	Mother or housemaid	
Mixed methods	Yes	Paper, Plastic, Glass, Textile, and Metal	Mother or housemaid	
Plastic bag	Yes	Battery and electronic waste	Father	
Plastic bag	No	0.00		0.00
Plastic bag	Yes	Plastic and Glass	Mother or housemaid	
Plastic bag	No	0.00		0.00
Mixed methods	No	0.00		0.00
Plastic bag	No	0.00		0.00
Plastic bag	Yes	Plastic and Glass	Mother or housemaid	
Plastic bag	No	0.00		0.00
Mixed methods	Yes	Paper, Plastic, Glass, Textile, and Metal	Mother or housemaid	
Plastic bag	No	0.00		0.00
Plastic bag	No	0.00		0.00
Plastic bag	No	0.00		0.00
Plastic bag	Yes	Plastic and Glass	Children	
Plastic bag	No	0.00		0.00
Plastic bag	Yes	Paper, Plastic, Glass, Textile, and Metal	Mother or housemaid	
Mixed methods	No	0.00		0.00

Plastic bag	Yes	Plastic and Glass	Mother or housemaid	
Plastic bag	No	0.00		0.00
Plastic bag	Yes	Plastic and Glass	Mother or housemaid	
Mixed methods	Yes	Battery and electronic waste	Father	
Plastic bag	Yes	Plastic and Glass	Mother or housemaid	
Open container	No	0.00		0.00
Plastic bag	Yes	Plastic and Glass	Mother or housemaid	
Plastic bag	No	0.00		0.00
Plastic bag	Yes	Plastic and Glass	Children	
Plastic bag	Yes	Battery and electronic waste	Father	
Plastic bag	Yes	Plastic and Glass	Mother or housemaid	
Plastic bag	Yes	Plastic and Glass	Mother or housemaid	
Plastic bag	Yes	Plastic and Glass	Mother or housemaid	
Plastic bag	Yes	Battery and electronic waste	Father	
Plastic bag	No	0.00		0.00
Plastic bag	Yes	Plastic and Glass	Mother or housemaid	
Plastic bag	No	0.00		0.00
Basket	Yes	Paper, Plastic, Glass, Textile, and Metal	Mother or housemaid	
Mixed methods	No	0.00		0.00
box	Yes	Plastic and Glass	Mother or housemaid	
Plastic bag	No	0.00		0.00
Plastic bag	No	0.00		0.00
Plastic bag	No	0.00		0.00
Plastic bag	Yes	Plastic and Glass	Mother or housemaid	
Plastic bag	No	0.00		0.00
Plastic bag	Yes	Plastic and Glass	Mother or housemaid	
Basket	No	0.00		0.00
Plastic bag	No	0.00		0.00
Plastic bag	No	0.00		0.00
Basket	Yes	Paper, Plastic, Glass, Textile, and Metal	Mother or housemaid	
Plastic bag	Yes	Plastic and Glass	Children	
Open container	Yes	Plastic and Glass	Mother or housemaid	
Plastic bag	No	0.00		0.00
Plastic bag	Yes	Plastic and Glass	Mother or housemaid	
Plastic bag	No	0.00		0.00
Plastic bag	Yes	Paper, Plastic, Glass, Textile, and Metal	Mother or housemaid	
Mixed methods	Yes	Plastic and Glass	Mother or housemaid	
box	No	0.00		0.00
Mixed methods	Yes	Paper, Plastic, Glass, Textile, and Metal	Mother or housemaid	
Plastic bag	Yes	Plastic and Glass	Children	
Mixed methods	Yes	Paper, Plastic, Glass, Textile, and Metal	Mother or housemaid	
Plastic bag	No	0.00		0.00
Open container	Yes	Plastic and Glass	Mother or housemaid	
Plastic bag	No	0.00		0.00
Mixed methods	Yes	Paper, Plastic, Glass, Textile, and Metal	Mother or housemaid	
Plastic bag	Yes	Plastic and Glass	Mother or housemaid	
Plastic bag	Yes	Paper, Plastic, Glass, Textile, and Metal	Mother or housemaid	
Mixed methods	Yes	Plastic and Glass	Children	

Plastic bag	Yes	Paper, Plastic, Glass, Textile, and Metal	Mother or housemaid	
Plastic bag	No	0.00		0.00
Open container	No	0.00		0.00
Mixed methods	No	0.00		0.00
Open container	Yes	Plastic and Glass	Mother or housemaid	
Plastic bag	Yes	Paper, Plastic, Glass, Textile, and Metal	All family member	
Plastic bag	Yes	Plastic and Glass	Mother or housemaid	
Plastic bag	Yes	Paper, Plastic, Glass, Textile, and Metal	Mother or housemaid	
Plastic bag	Yes	Plastic and Glass	Mother or housemaid	
Plastic bag	Yes	Paper, Plastic, Glass, Textile, and Metal	Mother or housemaid	
Plastic bag	Yes	Plastic and Glass	Mother or housemaid	
Plastic bag	Yes	Paper, Plastic, Glass, Textile, and Metal	All family member	
Plastic bag	Yes	Plastic and Glass	Mother or housemaid	

Dealing with recyclables	Reasons for not recycle
Sell	0.00
mix the recyclable materials with other materials	No enough recyclable materials
Own use	0.00
Own use	0.00
Sell	0.00
Sell	0.00
Own use	0.00
Sell	0.00
mix the recyclable materials with other materials	Not taking an interest
Sell	0.00
Own use	0.00
Sell	0.00
mix the recyclable materials with other materials	Having no time
Own use	0.00
mix the recyclable materials with other materials	Not taking an interest
mix the recyclable materials with other materials	Having no time
mix the recyclable materials with other materials	No enough recyclable materials
Sell	0.00
Give away	0.00
Own use	0.00
mix the recyclable materials with other materials	Not taking an interest
mix the recyclable materials with other materials	Having no time
Give away	0.00
Sell	0.00
Own use	0.00
Sell	0.00
Give away	0.00
Own use	0.00
Give away	0.00
Sell	0.00
Own use	0.00
mix the recyclable materials with other materials	Not taking an interest
mix the recyclable materials with other materials	Having no time
Own use	0.00
mix the recyclable materials with other materials	Lack of awareness
Sell	0.00
Own use	0.00
mix the recyclable materials with other materials	Not taking an interest
mix the recyclable materials with other materials	Lack of awareness
Own use	0.00
mix the recyclable materials with other materials	Lack of awareness
mix the recyclable materials with other materials	Not taking an interest
Sell	0.00
Give away	0.00
Own use	0.00
Sell	0.00
Own use	0.00

Sell		0.00
mix the recyclable materials with other materials	Having no time	
Own use		0.00
mix the recyclable materials with other materials	Having no time	
Give away		0.00
Own use		0.00
Own use		0.00
Sell		0.00
Own use		0.00
mix the recyclable materials with other materials	Having no time	
mix the recyclable materials with other materials	Not taking an interest	
mix the recyclable materials with other materials	Lack of awareness	
Sell		0.00
Own use		0.00
Sell		0.00
Give away		0.00
Own use		0.00
Own use		0.00
Own use		0.00
Own use		0.00
Own use		0.00
mix the recyclable materials with other materials	Not taking an interest	
mix the recyclable materials with other materials	Lack of awareness	
Own use		0.00
Give away		0.00
mix the recyclable materials with other materials	Lack of awareness	
mix the recyclable materials with other materials	Not taking an interest	
Own use		0.00
Own use		0.00
Own use		0.00
Own use		0.00
mix the recyclable materials with other materials	No enough recyclable materials	
Own use		0.00
mix the recyclable materials with other materials	Not taking an interest	
mix the recyclable materials with other materials	Having no time	
mix the recyclable materials with other materials	No enough recyclable materials	
Own use		0.00
Own use		0.00
Sell		0.00
Own use		0.00
Sell		0.00
Own use		0.00
Own use		0.00
Own use		0.00
Own use		0.00
mix the recyclable materials with other materials	No enough recyclable materials	
Sell		0.00
Own use		0.00

Sell		0.00
mix the recyclable materials with other materials	Not taking an interest	
Own use		0.00
mix the recyclable materials with other materials	Lack of awareness	
mix the recyclable materials with other materials	Having no time	
mix the recyclable materials with other materials	No enough recyclable materials	
Sell		0.00
Own use		0.00
Own use		0.00
mix the recyclable materials with other materials	Not taking an interest	
mix the recyclable materials with other materials	Having no time	
Sell		0.00
Sell		0.00
Own use		0.00
Sell		0.00
Own use		0.00
Own use		0.00
Own use		0.00
mix the recyclable materials with other materials	Not taking an interest	
Own use		0.00
mix the recyclable materials with other materials	Lack of awareness	
Give away		0.00
Sell		0.00
Give away		0.00
Give away		0.00
Own use		0.00
mix the recyclable materials with other materials	Not taking an interest	
mix the recyclable materials with other materials	Lack of awareness	
mix the recyclable materials with other materials	Lack of awareness	
Own use		0.00
Give away		0.00
Sell		0.00
Own use		0.00
Give away		0.00
Give away		0.00
Own use		0.00
Own use		0.00
Own use		0.00
mix the recyclable materials with other materials	Not taking an interest	
mix the recyclable materials with other materials	Lack of awareness	
Sell		0.00
Own use		0.00
Sell		0.00
mix the recyclable materials with other materials	Lack of awareness	
Own use		0.00
mix the recyclable materials with other materials	Not taking an interest	
Own use		0.00
Sell		0.00

Sell		0.00
mix the recyclable materials with other materials	Lack of awareness	
mix the recyclable materials with other materials	Lack of awareness	
Give away		0.00
Give away		0.00
Give away		0.00
Own use		0.00
Sell		0.00
mix the recyclable materials with other materials	Having no time	
Give away		0.00
mix the recyclable materials with other materials	Having no time	
mix the recyclable materials with other materials	Not taking an interest	
mix the recyclable materials with other materials	No enough recyclable materials	
Own use		0.00
Give away		0.00
Sell		0.00
Sell		0.00
Give away		0.00
Own use		0.00
Give away		0.00
mix the recyclable materials with other materials	Not taking an interest	
Own use		0.00
Own use		0.00
Sell		0.00
Own use		0.00
Give away		0.00
Own use		0.00
mix the recyclable materials with other materials	No enough recyclable materials	
Own use		0.00
Own use		0.00
Own use		0.00
mix the recyclable materials with other materials	Not taking an interest	
Give away		0.00
mix the recyclable materials with other materials	Having no time	
mix the recyclable materials with other materials	Lack of awareness	
mix the recyclable materials with other materials	Lack of awareness	
Own use		0.00
Sell		0.00
Own use		0.00
mix the recyclable materials with other materials	Not taking an interest	
mix the recyclable materials with other materials	Having no time	
Give away		0.00
mix the recyclable materials with other materials	Lack of awareness	
Give away		0.00
mix the recyclable materials with other materials	Having no time	
mix the recyclable materials with other materials	Not taking an interest	
Give away		0.00
Sell		0.00

Give away		0.00
mix the recyclable materials with other materials	No enough recyclable materials	
mix the recyclable materials with other materials	Lack of awareness	
Own use		0.00
mix the recyclable materials with other materials	Lack of awareness	
Give away		0.00
Own use		0.00
Give away		0.00
mix the recyclable materials with other materials	Lack of awareness	
Own use		0.00
mix the recyclable materials with other materials	Lack of awareness	
mix the recyclable materials with other materials	Not taking an interest	
mix the recyclable materials with other materials	No enough recyclable materials	
Sell		0.00
Give away		0.00
Own use		0.00
Sell		0.00
Own use		0.00
Own use		0.00
mix the recyclable materials with other materials	Lack of awareness	
Own use		0.00
Own use		0.00
mix the recyclable materials with other materials	Lack of awareness	
mix the recyclable materials with other materials	Lack of awareness	
Own use		0.00
Sell		0.00
mix the recyclable materials with other materials	Not taking an interest	
mix the recyclable materials with other materials	Lack of awareness	
Own use		0.00
mix the recyclable materials with other materials	Lack of awareness	
Own use		0.00
Own use		0.00
Sell		0.00
mix the recyclable materials with other materials	Not taking an interest	
Give away		0.00
mix the recyclable materials with other materials	Lack of awareness	
mix the recyclable materials with other materials	Having no time	
mix the recyclable materials with other materials	Lack of awareness	
Give away		0.00
mix the recyclable materials with other materials	Lack of awareness	
Own use		0.00
mix the recyclable materials with other materials	Lack of awareness	
mix the recyclable materials with other materials	Having no time	
mix the recyclable materials with other materials	Not taking an interest	
Own use		0.00
mix the recyclable materials with other materials	Lack of awareness	
Own use		0.00
mix the recyclable materials with other materials	Lack of awareness	

Own use		0.00
mix the recyclable materials with other materials	Not taking an interest	
Own use		0.00
Own use		0.00
Sell		0.00
mix the recyclable materials with other materials	Having no time	
Sell		0.00
mix the recyclable materials with other materials	Lack of awareness	
Sell		0.00
Sell		0.00
Own use		0.00
Own use		0.00
Sell		0.00
Sell		0.00
mix the recyclable materials with other materials	Not taking an interest	
Give away		0.00
mix the recyclable materials with other materials	Lack of awareness	
Sell		0.00
mix the recyclable materials with other materials	Having no time	
Sell		0.00
mix the recyclable materials with other materials	Lack of awareness	
mix the recyclable materials with other materials	Having no time	
mix the recyclable materials with other materials	Lack of awareness	
Own use		0.00
mix the recyclable materials with other materials	Lack of awareness	
Sell		0.00
mix the recyclable materials with other materials	Lack of awareness	
mix the recyclable materials with other materials	Having no time	
mix the recyclable materials with other materials	Lack of awareness	
Give away		0.00
Give away		0.00
Give away		0.00
mix the recyclable materials with other materials	No enough recyclable materials	
Give away		0.00
mix the recyclable materials with other materials	Not taking an interest	
Give away		0.00
Own use		0.00
mix the recyclable materials with other materials	Not taking an interest	
Give away		0.00
Give away		0.00
Sell		0.00
mix the recyclable materials with other materials	Lack of awareness	
Sell		0.00
mix the recyclable materials with other materials	Lack of awareness	
Give away		0.00
Own use		0.00
Give away		0.00
Give away		0.00

Sell		0.00
mix the recyclable materials with other materials	Not taking an interest	
mix the recyclable materials with other materials	Lack of awareness	
mix the recyclable materials with other materials	Lack of awareness	
Own use		0.00
Sell		0.00
Own use		0.00
Own use		0.00
Give away		0.00
Give away		0.00
Own use		0.00
Sell		0.00
Own use		0.00

Reasons for not compost	Availability of collection service	Frequency of collection service
No time for these practices	No	No service
Do not know how to compost	Yes	three times per week
No land available	Yes	three times per week
No time for these practices	No	No service
No land available	No	No service
No land available	No	No service
No time for these practices	No	No service
No land available	Yes	every week
Do not know how to compost	Yes	three times per week
No time for these practices	Yes	three times per week
No land available	Yes	every week
No land available	No	No service
Do not know how to compost	No	No service
dirty and unhealthy	No	No service
No land available	Yes	every week
No time for these practices	Yes	three times per week
No land available	Yes	three times per week
Do not know how to compost	Yes	three times per week
No land available	Yes	every week
dirty and unhealthy	Yes	three times per week
No practical purpose for compost	Yes	three times per week
Do not know how to compost	Yes	every week
No practical purpose for compost	Yes	every week
No practical purpose for compost	Yes	three times per week
No time for these practices	No	No service
No land available	Yes	every week
No land available	Yes	three times per week
No land available	Yes	three times per week
dirty and unhealthy	Yes	three times per week
No land available	No	No service
Do not know how to compost	Yes	every week
No land available	Yes	three times per week
No land available	Yes	three times per week
dirty and unhealthy	Yes	every week
No land available	Yes	twice per week
No land available	Yes	twice per week
No time for these practices	No	No service
No land available	Yes	three times per week
No practical purpose for compost	Yes	three times per week
Do not know how to compost	Yes	three times per week
No land available	Yes	three times per week
No land available	Yes	three times per week
dirty and unhealthy	Yes	every week
No land available	Yes	twice per week
No land available	Yes	every day
No land available	Yes	twice per week
Do not know how to compost	No	No service

No land available	Yes	every day
No land available	Yes	three times per week
dirty and unhealthy	Yes	twice per week
No time for these practices	Yes	every day
No land available	No	No service
No land available	No	No service
No land available	Yes	every day
No land available	Yes	three times per week
dirty and unhealthy	Yes	every day
No land available	Yes	three times per week
No practical purpose for compost	No	No service
No practical purpose for compost	Yes	twice per week
No practical purpose for compost	Yes	three times per week
No practical purpose for compost	Yes	three times per week
dirty and unhealthy	Yes	three times per week
dirty and unhealthy	Yes	twice per week
dirty and unhealthy	No	No service
No land available	Yes	three times per week
No land available	Yes	three times per week
No land available	Yes	three times per week
No land available	Yes	three times per week
dirty and unhealthy	Yes	every day
No land available	Yes	three times per week
No land available	Yes	twice per week
dirty and unhealthy	Yes	twice per week
No land available	Yes	three times per week
No time for these practices	Yes	three times per week
No land available	Yes	three times per week
No land available	Yes	three times per week
Do not know how to compost	No	No service
No land available	Yes	three times per week
No land available	Yes	three times per week
Do not know how to compost	Yes	three times per week
No land available	No	No service
No land available	Yes	three times per week
dirty and unhealthy	No	No service
No land available	No	No service
No land available	Yes	every week
No land available	Yes	every week
No land available	Yes	every week
No land available	Yes	three times per week
No land available	Yes	three times per week
No time for these practices	Yes	three times per week
No land available	Yes	three times per week
Do not know how to compost	Yes	three times per week
No land available	Yes	every week
dirty and unhealthy	Yes	twice per week
No practical purpose for compost	No	No service

No land available	No	No service
No practical purpose for compost	Yes	every week
dirty and unhealthy	Yes	twice per week
No land available	Yes	three times per week
Do not know how to compost	Yes	three times per week
No land available	Yes	three times per week
No land available	Yes	three times per week
No land available	Yes	every day
dirty and unhealthy	Yes	three times per week
dirty and unhealthy	No	No service
No land available	Yes	twice per week
No land available	No	No service
No time for these practices	Yes	every week
No land available	Yes	three times per week
dirty and unhealthy	Yes	three times per week
No land available	No	No service
No land available	Yes	every day
No land available	Yes	every day
dirty and unhealthy	Yes	every day
No land available	No	No service
dirty and unhealthy	Yes	every day
No land available	Yes	three times per week
No practical purpose for compost	Yes	three times per week
dirty and unhealthy	Yes	three times per week
No land available	Yes	twice per week
No time for these practices	Yes	twice per week
No practical purpose for compost	Yes	three times per week
No land available	No	No service
Do not know how to compost	Yes	every day
No land available	Yes	three times per week
No time for these practices	Yes	three times per week
dirty and unhealthy	Yes	twice per week
No practical purpose for compost	No	No service
No practical purpose for compost	Yes	twice per week
No land available	Yes	twice per week
dirty and unhealthy	No	No service
dirty and unhealthy	No	No service
No land available	Yes	three times per week
No land available	Yes	three times per week
No time for these practices	Yes	three times per week
No land available	Yes	three times per week
dirty and unhealthy	Yes	three times per week
No land available	Yes	twice per week
Do not know how to compost	Yes	twice per week
dirty and unhealthy	Yes	twice per week
No land available	Yes	every day
No land available	Yes	three times per week
dirty and unhealthy	Yes	three times per week

No land available	Yes	three times per week
No land available	Yes	three times per week
No land available	Yes	twice per week
No land available	Yes	twice per week
No time for these practices	Yes	three times per week
No land available	Yes	three times per week
No practical purpose for compost	Yes	three times per week
No practical purpose for compost	Yes	three times per week
No practical purpose for compost	No	No service
No land available	Yes	every day
No land available	No	No service
No land available	Yes	three times per week
No land available	Yes	three times per week
No land available	Yes	three times per week
No land available	Yes	three times per week
No land available	Yes	three times per week
No land available	Yes	three times per week
No practical purpose for compost	Yes	three times per week
No land available	Yes	three times per week
Do not know how to compost	Yes	three times per week
Do not know how to compost	No	No service
No land available	No	No service
No practical purpose for compost	No	No service
No land available	Yes	every week
No time for these practices	Yes	every day
No land available	Yes	three times per week
Do not know how to compost	Yes	three times per week
No practical purpose for compost	Yes	twice per week
No practical purpose for compost	No	No service
Do not know how to compost	Yes	twice per week
No land available	Yes	three times per week
No land available	Yes	three times per week
No land available	Yes	three times per week
Do not know how to compost	Yes	three times per week
No land available	Yes	every week
Do not know how to compost	Yes	twice per week
No land available	No	No service
No land available	Yes	every day
No time for these practices	Yes	three times per week
No land available	Yes	three times per week
Do not know how to compost	Yes	three times per week
No land available	Yes	three times per week
No land available	Yes	three times per week
Do not know how to compost	Yes	twice per week
No land available	No	No service
Do not know how to compost	Yes	twice per week
No practical purpose for compost	Yes	every day
No practical purpose for compost	Yes	twice per week

No time for these practices	Yes	three times per week
No land available	Yes	three times per week
No land available	Yes	three times per week
No land available	Yes	three times per week
dirty and unhealthy	Yes	three times per week
Do not know how to compost	Yes	twice per week
No land available	Yes	three times per week
No land available	Yes	three times per week
No land available	Yes	three times per week
No land available	Yes	twice per week
No time for these practices	Yes	twice per week
No practical purpose for compost	Yes	three times per week
No practical purpose for compost	Yes	three times per week
dirty and unhealthy	Yes	three times per week
No land available	Yes	three times per week
Do not know how to compost	Yes	three times per week
No land available	Yes	every day
No practical purpose for compost	Yes	twice per week
dirty and unhealthy	Yes	every day
No land available	Yes	twice per week
No land available	Yes	every day
No time for these practices	Yes	twice per week
No land available	Yes	three times per week
No land available	Yes	three times per week
Do not know how to compost	Yes	three times per week
No land available	Yes	three times per week
No land available	Yes	twice per week
No land available	Yes	twice per week
No land available	Yes	every day
dirty and unhealthy	Yes	twice per week
No land available	Yes	every day
No land available	Yes	twice per week
No land available	Yes	three times per week
No land available	Yes	three times per week
No land available	Yes	three times per week
No land available	Yes	three times per week
No land available	Yes	three times per week
No time for these practices	Yes	three times per week
No land available	Yes	three times per week
Do not know how to compost	Yes	three times per week
No land available	Yes	three times per week
No land available	Yes	three times per week
No land available	Yes	three times per week
Do not know how to compost	Yes	three times per week
No land available	Yes	three times per week
No land available	Yes	three times per week
No land available	Yes	three times per week
No land available	Yes	every day

No land available	Yes	three times per week
Do not know how to compost	No	No service
No land available	Yes	every day
No land available	Yes	three times per week
No land available	Yes	three times per week
No land available	Yes	three times per week
No land available	Yes	three times per week
No time for these practices	Yes	three times per week
No land available	Yes	three times per week
No land available	Yes	three times per week
No land available	Yes	three times per week
No land available	Yes	every week
Do not know how to compost	No	No service
No land available	Yes	three times per week
No land available	Yes	three times per week
No land available	No	No service
No land available	Yes	three times per week
No time for these practices	Yes	three times per week
No land available	Yes	three times per week
No land available	Yes	three times per week
No land available	Yes	every week
Do not know how to compost	Yes	every week
No land available	Yes	every day
No time for these practices	No	No service
No land available	Yes	three times per week
No land available	Yes	every day
dirty and unhealthy	Yes	three times per week
No land available	Yes	three times per week
No land available	Yes	three times per week
No land available	Yes	every day
No time for these practices	Yes	every day
No land available	Yes	every day
Do not know how to compost	Yes	three times per week
No practical purpose for compost	Yes	three times per week
No land available	Yes	three times per week
No land available	Yes	three times per week
No practical purpose for compost	No	No service
No land available	Yes	every day
No land available	Yes	twice per week
No time for these practices	Yes	twice per week
No practical purpose for compost	Yes	three times per week
No practical purpose for compost	Yes	twice per week
dirty and unhealthy	Yes	three times per week
No land available	Yes	twice per week
No practical purpose for compost	Yes	twice per week
No land available	Yes	three times per week
No practical purpose for compost	Yes	three times per week
No practical purpose for compost	Yes	three times per week

dirty and unhealthy	Yes	three times per week
No practical purpose for compost	No	No service
No practical purpose for compost	Yes	twice per week
Do not know how to compost	Yes	twice per week
No land available	Yes	three times per week
No practical purpose for compost	Yes	twice per week
dirty and unhealthy	Yes	three times per week
No practical purpose for compost	Yes	three times per week
No time for these practices	Yes	three times per week
No practical purpose for compost	Yes	three times per week
No land available	Yes	three times per week
No practical purpose for compost	Yes	three times per week
dirty and unhealthy	No	No service

Alternative waste disposal way	waste management responsibility at house level	Organic	Plastic
Dumping on open spaces	Mother	0.78048	0.08780
0.00	Father	0.78835	0.08869
0.00	Children	0.86796	0.09765
Dumping on open spaces	Mother	0.74160	0.08343
Dumping on open spaces	Mother	1.30114	0.14638
Dumping on open spaces	Mother	1.30371	0.14667
Dumping on open spaces	Mother	1.29857	0.14609
0.00	Father	1.28829	0.14493
0.00	Mother	1.27800	0.14378
0.00	Children	1.28571	0.14464
0.00	Mother	1.31657	0.14811
Burning	Father	1.88743	0.21234
Burning	Mother	1.86943	0.21031
Dumping on open spaces	Mother	1.86429	0.20973
0.00	Mother	1.86943	0.21031
0.00	Mother	1.86943	0.21031
0.00	Mother	1.87200	0.21060
0.00	Father	2.49171	0.28032
0.00	Mother	2.53800	0.28553
0.00	Mother	2.52257	0.28379
0.00	Mother	2.50200	0.28148
0.00	Mother	2.52514	0.28408
0.00	Mother	2.50971	0.28234
0.00	Father	2.52257	0.28379
Dumping on open spaces	Children	2.55343	0.28726
0.00	Mother	2.52514	0.28408
0.00	Father	2.53286	0.28495
0.00	Housemaid	2.52000	0.28350
0.00	Mother	2.49686	0.28090
Burning	Mother	2.92886	0.32950
0.00	Mother	2.85377	0.32105
0.00	Mother	2.93503	0.33019
0.00	Mother	2.89080	0.32522
0.00	Mother	2.99211	0.33661
0.00	Mother	3.04097	0.34211
0.00	Mother	2.80851	0.31596
Dumping on open spaces	Mother	2.82806	0.31816
0.00	Mother	2.96794	0.33389
0.00	Mother	3.01629	0.33933
0.00	Mother	2.91600	0.32805
0.00	Mother	2.98440	0.33575
0.00	Mother	2.80440	0.31550
0.00	Mother	2.93863	0.33060
0.00	Mother	2.96331	0.33337
0.00	Housemaid	2.70771	0.30462
0.00	Mother	2.74371	0.30867
Dumping on open spaces	Mother	2.85686	0.32140

	0.00	Father	2.68971	0.30259
	0.00	Mother	2.87229	0.32313
	0.00	Mother	2.75914	0.31040
	0.00	Mother	2.81314	0.31648
Dumping on open spaces		Children	2.76943	0.31156
Dumping on open spaces		Mother	2.85429	0.32111
	0.00	Mother	2.87743	0.32371
	0.00	Father	2.85943	0.32169
	0.00	Mother	3.00857	0.33846
	0.00	Mother	3.20451	0.36051
Dumping on open spaces		Mother	3.15154	0.35455
	0.00	Mother	2.98311	0.33560
	0.00	Father	3.20914	0.36103
	0.00	Mother	3.24951	0.36557
	0.00	Children	3.20040	0.36005
	0.00	Mother	3.43826	0.38680
Burning		Mother	3.65143	0.41079
	0.00	Father	3.74451	0.42126
	0.00	Mother	3.48994	0.39262
	0.00	Children	3.42823	0.38568
	0.00	Father	3.83966	0.43196
	0.00	Mother	3.58766	0.40361
	0.00	Mother	3.85637	0.43384
	0.00	Mother	4.07057	0.45794
	0.00	Children	3.79286	0.42670
	0.00	Mother	4.13049	0.46468
	0.00	Mother	3.72909	0.41952
	0.00	Mother	2.35851	0.26533
	0.00	Father	0.76827	0.10280
Dumping on open spaces		Mother	0.76827	0.10280
	0.00	Mother	0.80443	0.10764
	0.00	Mother	0.77182	0.10327
	0.00	Housemaid	0.79647	0.10657
Dumping on open spaces		Mother	1.28490	0.17192
	0.00	Mother	1.28490	0.17192
Burning		Mother	1.28490	0.17192
Dumping on open spaces		Children	1.24843	0.16704
	0.00	Mother	1.24666	0.16681
	0.00	Mother	1.25569	0.16801
	0.00	Children	1.95676	0.26182
	0.00	Father	1.73179	0.23172
	0.00	Father	1.95676	0.26182
	0.00	Mother	1.71211	0.22909
	0.00	Mother	1.95625	0.26175
	0.00	Children	1.91330	0.25600
	0.00	Mother	1.90909	0.25544
	0.00	Mother	2.52567	0.33794
Dumping on open spaces		Mother	2.50021	0.33454

Dumping on open spaces		Father	2.51046	0.33591
	0.00	Mother	2.51249	0.33618
	0.00	Children	2.55159	0.34141
	0.00	Mother	2.51558	0.33659
	0.00	Mother	2.51036	0.33589
	0.00	Mother	2.52263	0.33754
	0.00	Mother	2.49347	0.33363
	0.00	Father	2.51543	0.33657
	0.00	Housemaid	2.57187	0.34412
Burying		Mother	2.94990	0.39470
	0.00	Mother	2.94990	0.39470
Dumping on open spaces		Children	3.03292	0.40581
	0.00	Father	3.03084	0.40553
	0.00	Children	3.02328	0.40452
	0.00	Mother	2.50158	0.33472
Dumping on open spaces		Mother	3.02780	0.40513
	0.00	Housemaid	3.03140	0.40561
	0.00	Mother	2.94990	0.39470
	0.00	Mother	2.52263	0.33754
Dumping on open spaces		Mother	2.50539	0.33523
	0.00	Mother	2.49007	0.33318
	0.00	Children	2.55159	0.34141
	0.00	Father	3.39644	0.45445
	0.00	Children	3.40430	0.45550
	0.00	Mother	3.48610	0.46645
	0.00	Children	3.47342	0.46475
	0.00	Mother	3.49066	0.46706
Dumping on open spaces		Mother	3.49371	0.46747
	0.00	Mother	3.51202	0.46992
	0.00	Father	3.52738	0.47197
	0.00	Mother	3.49112	0.46712
	0.00	Mother	3.47570	0.46506
Dumping on open spaces		Children	3.49295	0.46737
	0.00	Mother	3.47063	0.46438
	0.00	Mother	3.49589	0.46776
Dumping on open spaces		Mother	4.03584	0.54001
Dumping on open spaces		Mother	4.03204	0.53950
	0.00	Mother	4.03305	0.53963
	0.00	Mother	4.03534	0.53994
	0.00	Mother	4.02950	0.53916
	0.00	Mother	4.03103	0.53936
	0.00	Mother	4.60359	0.61597
	0.00	Mother	4.60156	0.61570
	0.00	Mother	4.60258	0.61584
	0.00	Mother	4.60080	0.61560
	0.00	Mother	4.59649	0.61502
	0.00	Mother	4.59776	0.61519
	0.00	Mother	4.60283	0.61587

	0.00	Mother	4.98268	0.66670
	0.00	Mother	5.02776	0.67273
	0.00	Mother	4.97604	0.66581
	0.00	Mother	4.88490	0.65361
	0.00	Mother	4.98973	0.66764
	0.00	Mother	5.05931	0.67695
	0.00	Mother	4.99434	0.66826
	0.00	Children	0.71852	0.13093
Dumping on open spaces		Mother	0.70867	0.12914
	0.00	Housemaid	0.72779	0.13262
Burning		Mother	0.71259	0.12985
	0.00	Mother	0.78996	0.14395
	0.00	Mother	1.08478	0.19767
	0.00	Children	0.98163	0.17888
	0.00	Housemaid	1.17564	0.21423
	0.00	Mother	1.17555	0.21421
	0.00	Mother	1.17722	0.21452
	0.00	Housemaid	1.18406	0.21577
	0.00	Children	1.62019	0.29524
	0.00	Mother	1.61128	0.29362
Burying		Mother	1.58845	0.28946
Dumping on open spaces		Mother	1.63204	0.29740
Dumping on open spaces		Mother	1.61077	0.29352
	0.00	Father	1.61770	0.29479
	0.00	Housemaid	1.60953	0.29330
	0.00	Mother	1.61378	0.29407
	0.00	Mother	1.60326	0.29216
	0.00	Mother	2.04202	0.37211
Burning		Mother	2.37873	0.43347
	0.00	Mother	2.34325	0.42700
	0.00	Housemaid	2.32254	0.42323
	0.00	Mother	2.36247	0.43050
	0.00	Children	2.34325	0.42700
	0.00	Mother	2.32905	0.42441
	0.00	Mother	2.35482	0.42911
	0.00	Mother	2.34803	0.42787
Dumping on open spaces		Mother	2.31107	0.42114
	0.00	Housemaid	3.14412	0.57294
	0.00	Mother	3.29117	0.59973
	0.00	Mother	3.28328	0.59830
	0.00	Mother	3.29552	0.60053
	0.00	Children	3.29863	0.60109
	0.00	Mother	3.31575	0.60421
	0.00	Mother	2.93294	0.53446
Dumping on open spaces		Mother	3.29490	0.60041
	0.00	Mother	2.90750	0.52982
	0.00	Housemaid	3.29614	0.60064
	0.00	Mother	3.27548	0.59688

0.00	Mother	3.29858	0.60109
0.00	Mother	2.93294	0.53446
0.00	Mother	2.88669	0.52603
0.00	Mother	2.89984	0.52843
0.00	Mother	2.90750	0.52982
0.00	Mother	3.80563	0.69348
0.00	Mother	3.80204	0.69283
0.00	Mother	3.80300	0.69300
0.00	Mother	3.80515	0.69340
0.00	Children	3.79965	0.69239
0.00	Mother	3.80109	0.69266
0.00	Mother	3.57035	0.65061
0.00	Mother	3.33602	0.60791
0.00	Father	3.80204	0.69283
0.00	Mother	3.80300	0.69300
0.00	Mother	3.80515	0.69340
0.00	Mother	3.91815	0.71399
0.00	Mother	3.80563	0.69348
0.00	Mother	4.47154	0.81483
0.00	Mother	4.47250	0.81500
0.00	Mother	4.47465	0.81540
0.00	Mother	4.46915	0.81439
0.00	Mother	4.33430	0.78982
0.00	Mother	4.33549	0.79004
0.00	Father	4.34027	0.79091
0.00	Mother	5.00786	0.91256
0.00	Mother	5.00380	0.91182
0.00	Mother	5.00499	0.91204
0.00	Mother	5.00977	0.91291
0.00	Mother	4.74604	0.86485
0.00	Mother	5.45523	0.99408
0.00	Mother	5.42233	0.98809
0.00	Mother	5.44471	0.99217
0.00	Mother	5.37895	0.98018
0.00	Housemaid	0.78023	0.14817
0.00	Children	0.75175	0.14276
0.00	Mother	0.71795	0.13634
0.00	Mother	0.76900	0.14603
0.00	Housemaid	0.76544	0.14536
0.00	Mother	1.37046	0.26025
0.00	Mother	1.37250	0.26064
0.00	Mother	1.37648	0.26140
0.00	Mother	1.36719	0.25963
0.00	Mother	1.39900	0.26567
0.00	Children	1.20075	0.22802
0.00	Mother	1.78799	0.33954
0.00	Housemaid	1.82861	0.34726
0.00	Mother	1.89486	0.35984

	0.00	Mother	1.83017	0.34755
Dumping on open spaces		Mother	1.83695	0.34884
	0.00	Housemaid	1.78406	0.33880
	0.00	Mother	1.89799	0.36043
	0.00	Mother	1.79292	0.34048
	0.00	Mother	1.82813	0.34717
	0.00	Mother	2.30173	0.43710
	0.00	Mother	2.35552	0.44732
	0.00	Children	2.75670	0.52350
	0.00	Children	2.33173	0.44280
	0.00	Mother	2.35742	0.44768
	0.00	Mother	2.33647	0.44370
Dumping on open spaces		Mother	2.34135	0.44463
	0.00	Father	2.38448	0.45282
	0.00	Mother	2.33775	0.44394
Dumping on open spaces		Mother	3.11594	0.59172
	0.00	Mother	3.26167	0.61940
	0.00	Mother	3.25385	0.61791
	0.00	Mother	3.26598	0.62022
	0.00	Mother	3.26906	0.62080
	0.00	Mother	3.28603	0.62402
	0.00	Mother	3.29802	0.62630
	0.00	Mother	3.26537	0.62010
Burning		Mother	3.25177	0.61752
	0.00	Mother	3.26660	0.62033
	0.00	Housemaid	3.24613	0.61645
	0.00	Mother	3.26902	0.62079
	0.00	Mother	2.90665	0.55198
	0.00	Mother	2.86082	0.54328
	0.00	Mother	2.87386	0.54575
	0.00	Children	2.88144	0.54719
	0.00	Housemaid	2.89859	0.55045
	0.00	Mother	3.58029	0.67991
	0.00	Mother	3.58195	0.68022
	0.00	Mother	3.55195	0.67452
	0.00	Mother	3.62233	0.68789
Dumping on open spaces		Mother	3.50285	0.66520
	0.00	Mother	3.52252	0.66893
	0.00	Mother	3.49096	0.66294
	0.00	Children	3.30613	0.62784
	0.00	Housemaid	3.55987	0.67603
	0.00	Mother	3.49451	0.66362
	0.00	Mother	3.63655	0.69059
	0.00	Mother	3.49975	0.66461
	0.00	Mother	3.54233	0.67270
	0.00	Mother	4.04645	0.76843
	0.00	Mother	4.05526	0.77010
	0.00	Children	4.03948	0.76711

	0.00	Housemaid	4.17408	0.79267
Dumping on open spaces		Mother	4.22934	0.80316
	0.00	Mother	4.21792	0.80099
	0.00	Mother	4.23029	0.80334
	0.00	Mother	4.29948	0.81648
	0.00	Mother	4.83094	0.91741
	0.00	Mother	4.88274	0.92724
	0.00	Mother	4.86564	0.92399
	0.00	Mother	4.71327	0.89506
	0.00	Mother	5.42293	1.02983
	0.00	Housemaid	5.74501	1.09099
	0.00	Father	5.41961	1.02920
Dumping on open spaces		Mother	5.44084	1.03323

Inert	Paper	Textile	Metal	Glass	Wood	Hazardous
0.09756	0.03902	0.02818	0.01409	0.01192	0.00650	0.01734
0.09854	0.03942	0.02847	0.01423	0.01204	0.00657	0.01752
0.10850	0.04340	0.03134	0.01567	0.01326	0.00723	0.01929
0.09270	0.03708	0.02678	0.01339	0.01133	0.00618	0.01648
0.16264	0.06506	0.04699	0.02349	0.01988	0.01084	0.02891
0.16296	0.06519	0.04708	0.02354	0.01992	0.01086	0.02897
0.16232	0.06493	0.04689	0.02345	0.01984	0.01082	0.02886
0.16104	0.06441	0.04652	0.02326	0.01968	0.01074	0.02863
0.15975	0.06390	0.04615	0.02308	0.01953	0.01065	0.02840
0.16071	0.06429	0.04643	0.02321	0.01964	0.01071	0.02857
0.16457	0.06583	0.04754	0.02377	0.02011	0.01097	0.02926
0.23593	0.09437	0.06816	0.03408	0.02884	0.01573	0.04194
0.23368	0.09347	0.06751	0.03375	0.02856	0.01558	0.04154
0.23304	0.09321	0.06732	0.03366	0.02848	0.01554	0.04143
0.23368	0.09347	0.06751	0.03375	0.02856	0.01558	0.04154
0.23368	0.09347	0.06751	0.03375	0.02856	0.01558	0.04154
0.23400	0.09360	0.06760	0.03380	0.02860	0.01560	0.04160
0.31146	0.12459	0.08998	0.04499	0.03807	0.02076	0.05537
0.31725	0.12690	0.09165	0.04583	0.03878	0.02115	0.05640
0.31532	0.12613	0.09109	0.04555	0.03854	0.02102	0.05606
0.31275	0.12510	0.09035	0.04518	0.03823	0.02085	0.05560
0.31564	0.12626	0.09119	0.04559	0.03858	0.02104	0.05611
0.31371	0.12549	0.09063	0.04531	0.03834	0.02091	0.05577
0.31532	0.12613	0.09109	0.04555	0.03854	0.02102	0.05606
0.31918	0.12767	0.09221	0.04610	0.03901	0.02128	0.05674
0.31564	0.12626	0.09119	0.04559	0.03858	0.02104	0.05611
0.31661	0.12664	0.09146	0.04573	0.03870	0.02111	0.05629
0.31500	0.12600	0.09100	0.04550	0.03850	0.02100	0.05600
0.31211	0.12484	0.09016	0.04508	0.03815	0.02081	0.05549
0.36611	0.14644	0.10576	0.05288	0.04475	0.02441	0.06509
0.35672	0.14269	0.10305	0.05153	0.04360	0.02378	0.06342
0.36688	0.14675	0.10599	0.05299	0.04484	0.02446	0.06522
0.36135	0.14454	0.10439	0.05220	0.04417	0.02409	0.06424
0.37401	0.14961	0.10805	0.05402	0.04571	0.02493	0.06649
0.38012	0.15205	0.10981	0.05491	0.04646	0.02534	0.06758
0.35106	0.14043	0.10142	0.05071	0.04291	0.02340	0.06241
0.35351	0.14140	0.10212	0.05106	0.04321	0.02357	0.06285
0.37099	0.14840	0.10718	0.05359	0.04534	0.02473	0.06595
0.37704	0.15081	0.10892	0.05446	0.04608	0.02514	0.06703
0.36450	0.14580	0.10530	0.05265	0.04455	0.02430	0.06480
0.37305	0.14922	0.10777	0.05389	0.04560	0.02487	0.06632
0.35055	0.14022	0.10127	0.05064	0.04285	0.02337	0.06232
0.36733	0.14693	0.10612	0.05306	0.04490	0.02449	0.06530
0.37041	0.14817	0.10701	0.05350	0.04527	0.02469	0.06585
0.33846	0.13539	0.09778	0.04889	0.04137	0.02256	0.06017
0.34296	0.13719	0.09908	0.04954	0.04192	0.02286	0.06097
0.35711	0.14284	0.10316	0.05158	0.04365	0.02381	0.06349

0.33621	0.13449	0.09713	0.04856	0.04109	0.02241	0.05977
0.35904	0.14361	0.10372	0.05186	0.04388	0.02394	0.06383
0.34489	0.13796	0.09964	0.04982	0.04215	0.02299	0.06131
0.35164	0.14066	0.10159	0.05079	0.04298	0.02344	0.06251
0.34618	0.13847	0.10001	0.05000	0.04231	0.02308	0.06154
0.35679	0.14271	0.10307	0.05154	0.04361	0.02379	0.06343
0.35968	0.14387	0.10391	0.05195	0.04396	0.02398	0.06394
0.35743	0.14297	0.10326	0.05163	0.04369	0.02383	0.06354
0.37607	0.15043	0.10864	0.05432	0.04596	0.02507	0.06686
0.40056	0.16023	0.11572	0.05786	0.04896	0.02670	0.07121
0.39394	0.15758	0.11381	0.05690	0.04815	0.02626	0.07003
0.37289	0.14916	0.10772	0.05386	0.04558	0.02486	0.06629
0.40114	0.16046	0.11589	0.05794	0.04903	0.02674	0.07131
0.40619	0.16248	0.11734	0.05867	0.04965	0.02708	0.07221
0.40005	0.16002	0.11557	0.05779	0.04890	0.02667	0.07112
0.42978	0.17191	0.12416	0.06208	0.05253	0.02865	0.07641
0.45643	0.18257	0.13186	0.06593	0.05579	0.03043	0.08114
0.46806	0.18723	0.13522	0.06761	0.05721	0.03120	0.08321
0.43624	0.17450	0.12603	0.06301	0.05332	0.02908	0.07755
0.42853	0.17141	0.12380	0.06190	0.05238	0.02857	0.07618
0.47996	0.19198	0.13865	0.06933	0.05866	0.03200	0.08533
0.44846	0.17938	0.12955	0.06478	0.05481	0.02990	0.07973
0.48205	0.19282	0.13926	0.06963	0.05892	0.03214	0.08570
0.50882	0.20353	0.14699	0.07350	0.06219	0.03392	0.09046
0.47411	0.18964	0.13696	0.06848	0.05795	0.03161	0.08429
0.51631	0.20652	0.14916	0.07458	0.06310	0.03442	0.09179
0.46614	0.18645	0.13466	0.06733	0.05697	0.03108	0.08287
0.29481	0.11793	0.08517	0.04258	0.03603	0.01965	0.05241
0.09414	0.04328	0.02597	0.01298	0.01190	0.00649	0.01731
0.09414	0.04328	0.02597	0.01298	0.01190	0.00649	0.01731
0.09857	0.04532	0.02719	0.01360	0.01246	0.00680	0.01813
0.09458	0.04348	0.02609	0.01304	0.01196	0.00652	0.01739
0.09760	0.04487	0.02692	0.01346	0.01234	0.00673	0.01795
0.15745	0.07239	0.04343	0.02172	0.01991	0.01086	0.02896
0.15745	0.07239	0.04343	0.02172	0.01991	0.01086	0.02896
0.15745	0.07239	0.04343	0.02172	0.01991	0.01086	0.02896
0.15298	0.07033	0.04220	0.02110	0.01934	0.01055	0.02813
0.15276	0.07023	0.04214	0.02107	0.01931	0.01054	0.02809
0.15387	0.07074	0.04245	0.02122	0.01945	0.01061	0.02830
0.23977	0.11024	0.06614	0.03307	0.03032	0.01654	0.04410
0.21221	0.09757	0.05854	0.02927	0.02683	0.01463	0.03903
0.23977	0.11024	0.06614	0.03307	0.03032	0.01654	0.04410
0.20979	0.09646	0.05787	0.02894	0.02653	0.01447	0.03858
0.23971	0.11021	0.06613	0.03306	0.03031	0.01653	0.04408
0.23445	0.10779	0.06467	0.03234	0.02964	0.01617	0.04312
0.23393	0.10755	0.06453	0.03227	0.02958	0.01613	0.04302
0.30948	0.14229	0.08537	0.04269	0.03913	0.02134	0.05692
0.30636	0.14086	0.08451	0.04226	0.03874	0.02113	0.05634

0.30762	0.14143	0.08486	0.04243	0.03889	0.02122	0.05657
0.30787	0.14155	0.08493	0.04246	0.03893	0.02123	0.05662
0.31266	0.14375	0.08625	0.04313	0.03953	0.02156	0.05750
0.30825	0.14172	0.08503	0.04252	0.03897	0.02126	0.05669
0.30761	0.14143	0.08486	0.04243	0.03889	0.02121	0.05657
0.30911	0.14212	0.08527	0.04264	0.03908	0.02132	0.05685
0.30554	0.14048	0.08429	0.04214	0.03863	0.02107	0.05619
0.30823	0.14171	0.08503	0.04251	0.03897	0.02126	0.05669
0.31515	0.14489	0.08694	0.04347	0.03985	0.02173	0.05796
0.36147	0.16619	0.09971	0.04986	0.04570	0.02493	0.06648
0.36147	0.16619	0.09971	0.04986	0.04570	0.02493	0.06648
0.37164	0.17087	0.10252	0.05126	0.04699	0.02563	0.06835
0.37138	0.17075	0.10245	0.05123	0.04696	0.02561	0.06830
0.37046	0.17033	0.10220	0.05110	0.04684	0.02555	0.06813
0.30653	0.14093	0.08456	0.04228	0.03876	0.02114	0.05637
0.37101	0.17058	0.10235	0.05117	0.04691	0.02559	0.06823
0.37145	0.17078	0.10247	0.05123	0.04697	0.02562	0.06831
0.36147	0.16619	0.09971	0.04986	0.04570	0.02493	0.06648
0.30911	0.14212	0.08527	0.04264	0.03908	0.02132	0.05685
0.30700	0.14115	0.08469	0.04234	0.03882	0.02117	0.05646
0.30512	0.14029	0.08417	0.04209	0.03858	0.02104	0.05611
0.31266	0.14375	0.08625	0.04313	0.03953	0.02156	0.05750
0.41618	0.19135	0.11481	0.05740	0.05262	0.02870	0.07654
0.41715	0.19179	0.11507	0.05754	0.05274	0.02877	0.07672
0.42717	0.19640	0.11784	0.05892	0.05401	0.02946	0.07856
0.42562	0.19569	0.11741	0.05871	0.05381	0.02935	0.07827
0.42773	0.19666	0.11799	0.05900	0.05408	0.02950	0.07866
0.42810	0.19683	0.11810	0.05905	0.05413	0.02952	0.07873
0.43035	0.19786	0.11872	0.05936	0.05441	0.02968	0.07914
0.43223	0.19873	0.11924	0.05962	0.05465	0.02981	0.07949
0.42779	0.19668	0.11801	0.05900	0.05409	0.02950	0.07867
0.42590	0.19581	0.11749	0.05874	0.05385	0.02937	0.07833
0.42801	0.19679	0.11807	0.05904	0.05412	0.02952	0.07871
0.42527	0.19553	0.11732	0.05866	0.05377	0.02933	0.07821
0.42837	0.19695	0.11817	0.05909	0.05416	0.02954	0.07878
0.49453	0.22737	0.13642	0.06821	0.06253	0.03411	0.09095
0.49407	0.22716	0.13629	0.06815	0.06247	0.03407	0.09086
0.49419	0.22721	0.13633	0.06816	0.06248	0.03408	0.09089
0.49447	0.22734	0.13641	0.06820	0.06252	0.03410	0.09094
0.49376	0.22701	0.13621	0.06810	0.06243	0.03405	0.09081
0.49394	0.22710	0.13626	0.06813	0.06245	0.03407	0.09084
0.56410	0.25936	0.15561	0.07781	0.07132	0.03890	0.10374
0.56385	0.25924	0.15555	0.07777	0.07129	0.03889	0.10370
0.56398	0.25930	0.15558	0.07779	0.07131	0.03890	0.10372
0.56376	0.25920	0.15552	0.07776	0.07128	0.03888	0.10368
0.56323	0.25896	0.15537	0.07769	0.07121	0.03884	0.10358
0.56339	0.25903	0.15542	0.07771	0.07123	0.03885	0.10361
0.56401	0.25931	0.15559	0.07779	0.07131	0.03890	0.10373

0.61055	0.28071	0.16843	0.08421	0.07720	0.04211	0.11229
0.61608	0.28325	0.16995	0.08498	0.07789	0.04249	0.11330
0.60974	0.28034	0.16820	0.08410	0.07709	0.04205	0.11214
0.59857	0.27521	0.16512	0.08256	0.07568	0.04128	0.11008
0.61142	0.28111	0.16867	0.08433	0.07731	0.04217	0.11244
0.61994	0.28503	0.17102	0.08551	0.07838	0.04275	0.11401
0.61198	0.28137	0.16882	0.08441	0.07738	0.04221	0.11255
0.09230	0.05581	0.02737	0.01234	0.01234	0.00644	0.01717
0.09103	0.05504	0.02699	0.01217	0.01217	0.00635	0.01694
0.09349	0.05653	0.02772	0.01250	0.01250	0.00652	0.01739
0.09153	0.05535	0.02714	0.01224	0.01224	0.00639	0.01703
0.10147	0.06136	0.03009	0.01357	0.01357	0.00708	0.01888
0.13934	0.08425	0.04132	0.01863	0.01863	0.00972	0.02592
0.12609	0.07624	0.03739	0.01686	0.01686	0.00880	0.02346
0.15102	0.09131	0.04478	0.02019	0.02019	0.01054	0.02810
0.15100	0.09130	0.04477	0.02019	0.02019	0.01054	0.02809
0.15122	0.09143	0.04484	0.02022	0.02022	0.01055	0.02813
0.15210	0.09197	0.04510	0.02034	0.02034	0.01061	0.02830
0.20812	0.12584	0.06171	0.02783	0.02783	0.01452	0.03872
0.20698	0.12515	0.06137	0.02768	0.02768	0.01444	0.03851
0.20404	0.12337	0.06050	0.02728	0.02728	0.01424	0.03796
0.20964	0.12676	0.06216	0.02803	0.02803	0.01463	0.03900
0.20691	0.12511	0.06135	0.02767	0.02767	0.01444	0.03849
0.20780	0.12565	0.06162	0.02779	0.02779	0.01450	0.03866
0.20675	0.12501	0.06130	0.02765	0.02765	0.01442	0.03847
0.20730	0.12534	0.06147	0.02772	0.02772	0.01446	0.03857
0.20595	0.12453	0.06107	0.02754	0.02754	0.01437	0.03832
0.26231	0.15860	0.07778	0.03508	0.03508	0.01830	0.04880
0.30556	0.18476	0.09060	0.04086	0.04086	0.02132	0.05685
0.30100	0.18200	0.08925	0.04025	0.04025	0.02100	0.05600
0.29834	0.18039	0.08846	0.03989	0.03989	0.02081	0.05551
0.30347	0.18349	0.08998	0.04058	0.04058	0.02117	0.05646
0.30100	0.18200	0.08925	0.04025	0.04025	0.02100	0.05600
0.29918	0.18090	0.08871	0.04001	0.04001	0.02087	0.05566
0.30249	0.18290	0.08969	0.04045	0.04045	0.02110	0.05628
0.30161	0.18237	0.08943	0.04033	0.04033	0.02104	0.05611
0.29687	0.17950	0.08802	0.03970	0.03970	0.02071	0.05523
0.40387	0.24420	0.11975	0.05401	0.05401	0.02818	0.07514
0.42276	0.25562	0.12535	0.05653	0.05653	0.02950	0.07865
0.42175	0.25501	0.12505	0.05640	0.05640	0.02942	0.07847
0.42332	0.25596	0.12552	0.05661	0.05661	0.02953	0.07876
0.42372	0.25620	0.12564	0.05666	0.05666	0.02956	0.07883
0.42592	0.25753	0.12629	0.05695	0.05695	0.02972	0.07924
0.37675	0.22780	0.11171	0.05038	0.05038	0.02628	0.07009
0.42324	0.25591	0.12550	0.05660	0.05660	0.02953	0.07874
0.37348	0.22582	0.11074	0.04994	0.04994	0.02606	0.06948
0.42340	0.25601	0.12554	0.05662	0.05662	0.02954	0.07877
0.42075	0.25441	0.12476	0.05626	0.05626	0.02935	0.07828

0.42372	0.25620	0.12564	0.05666	0.05666	0.02956	0.07883
0.37675	0.22780	0.11171	0.05038	0.05038	0.02628	0.07009
0.37081	0.22421	0.10995	0.04958	0.04958	0.02587	0.06899
0.37250	0.22523	0.11045	0.04981	0.04981	0.02599	0.06930
0.37348	0.22582	0.11074	0.04994	0.04994	0.02606	0.06948
0.48885	0.29558	0.14495	0.06537	0.06537	0.03411	0.09095
0.48839	0.29530	0.14481	0.06531	0.06531	0.03407	0.09086
0.48851	0.29538	0.14485	0.06532	0.06532	0.03408	0.09089
0.48879	0.29555	0.14493	0.06536	0.06536	0.03410	0.09094
0.48808	0.29512	0.14472	0.06527	0.06527	0.03405	0.09081
0.48827	0.29523	0.14478	0.06529	0.06529	0.03407	0.09084
0.45863	0.27731	0.13599	0.06133	0.06133	0.03200	0.08533
0.42853	0.25911	0.12706	0.05730	0.05730	0.02990	0.07973
0.48839	0.29530	0.14481	0.06531	0.06531	0.03407	0.09086
0.48851	0.29538	0.14485	0.06532	0.06532	0.03408	0.09089
0.48879	0.29555	0.14493	0.06536	0.06536	0.03410	0.09094
0.50330	0.30432	0.14924	0.06730	0.06730	0.03511	0.09364
0.48885	0.29558	0.14495	0.06537	0.06537	0.03411	0.09095
0.57439	0.34730	0.17031	0.07681	0.07681	0.04007	0.10686
0.57451	0.34738	0.17035	0.07682	0.07682	0.04008	0.10689
0.57479	0.34755	0.17043	0.07686	0.07686	0.04010	0.10694
0.57408	0.34712	0.17022	0.07677	0.07677	0.04005	0.10681
0.55676	0.33664	0.16509	0.07445	0.07445	0.03884	0.10358
0.55691	0.33674	0.16513	0.07447	0.07447	0.03885	0.10361
0.55753	0.33711	0.16531	0.07455	0.07455	0.03890	0.10373
0.64328	0.38896	0.19074	0.08602	0.08602	0.04488	0.11968
0.64276	0.38864	0.19059	0.08595	0.08595	0.04484	0.11958
0.64291	0.38874	0.19063	0.08597	0.08597	0.04485	0.11961
0.64353	0.38911	0.19081	0.08605	0.08605	0.04490	0.11973
0.60965	0.36862	0.18077	0.08152	0.08152	0.04253	0.11342
0.70075	0.42371	0.20778	0.09370	0.09370	0.04889	0.13037
0.69652	0.42115	0.20653	0.09314	0.09314	0.04859	0.12959
0.69940	0.42289	0.20738	0.09352	0.09352	0.04880	0.13012
0.69095	0.41778	0.20487	0.09239	0.09239	0.04821	0.12855
0.09995	0.06585	0.02881	0.01352	0.01352	0.00706	0.01881
0.09631	0.06345	0.02776	0.01303	0.01303	0.00680	0.01813
0.09198	0.06060	0.02651	0.01244	0.01244	0.00649	0.01731
0.09852	0.06490	0.02840	0.01333	0.01333	0.00695	0.01854
0.09806	0.06460	0.02826	0.01327	0.01327	0.00692	0.01846
0.17557	0.11567	0.05060	0.02375	0.02375	0.01239	0.03305
0.17583	0.11584	0.05068	0.02379	0.02379	0.01241	0.03310
0.17634	0.11618	0.05083	0.02386	0.02386	0.01245	0.03319
0.17515	0.11539	0.05048	0.02370	0.02370	0.01236	0.03297
0.17922	0.11808	0.05166	0.02425	0.02425	0.01265	0.03374
0.15383	0.10134	0.04434	0.02081	0.02081	0.01086	0.02896
0.22906	0.15091	0.06602	0.03099	0.03099	0.01617	0.04312
0.23426	0.15434	0.06752	0.03169	0.03169	0.01654	0.04410
0.24275	0.15993	0.06997	0.03284	0.03284	0.01714	0.04569

0.23446	0.15447	0.06758	0.03172	0.03172	0.01655	0.04413
0.23533	0.15504	0.06783	0.03184	0.03184	0.01661	0.04430
0.22855	0.15058	0.06588	0.03092	0.03092	0.01613	0.04302
0.24315	0.16019	0.07008	0.03290	0.03290	0.01716	0.04577
0.22969	0.15132	0.06620	0.03108	0.03108	0.01621	0.04324
0.23420	0.15430	0.06750	0.03169	0.03169	0.01653	0.04408
0.29487	0.19427	0.08499	0.03989	0.03989	0.02081	0.05551
0.30176	0.19881	0.08698	0.04083	0.04083	0.02130	0.05680
0.35316	0.23267	0.10179	0.04778	0.04778	0.02493	0.06648
0.29871	0.19680	0.08610	0.04041	0.04041	0.02109	0.05623
0.30201	0.19897	0.08705	0.04086	0.04086	0.02132	0.05685
0.29932	0.19720	0.08628	0.04050	0.04050	0.02113	0.05634
0.29995	0.19761	0.08646	0.04058	0.04058	0.02117	0.05646
0.30547	0.20125	0.08805	0.04133	0.04133	0.02156	0.05750
0.29949	0.19731	0.08632	0.04052	0.04052	0.02114	0.05637
0.39918	0.26299	0.11506	0.05401	0.05401	0.02818	0.07514
0.41785	0.27529	0.12044	0.05653	0.05653	0.02950	0.07865
0.41685	0.27463	0.12015	0.05640	0.05640	0.02942	0.07847
0.41840	0.27565	0.12060	0.05661	0.05661	0.02953	0.07876
0.41880	0.27591	0.12071	0.05666	0.05666	0.02956	0.07883
0.42097	0.27734	0.12134	0.05695	0.05695	0.02972	0.07924
0.42250	0.27836	0.12178	0.05716	0.05716	0.02982	0.07953
0.41832	0.27560	0.12058	0.05660	0.05660	0.02953	0.07874
0.41658	0.27445	0.12007	0.05636	0.05636	0.02941	0.07841
0.41848	0.27570	0.12062	0.05662	0.05662	0.02954	0.07877
0.41586	0.27398	0.11986	0.05626	0.05626	0.02935	0.07828
0.41879	0.27591	0.12071	0.05666	0.05666	0.02956	0.07883
0.37237	0.24532	0.10733	0.05038	0.05038	0.02628	0.07009
0.36650	0.24146	0.10564	0.04958	0.04958	0.02587	0.06899
0.36817	0.24256	0.10612	0.04981	0.04981	0.02599	0.06930
0.36914	0.24320	0.10640	0.04994	0.04994	0.02606	0.06948
0.37133	0.24464	0.10703	0.05024	0.05024	0.02621	0.06990
0.45867	0.30218	0.13220	0.06205	0.06205	0.03238	0.08634
0.45888	0.30232	0.13227	0.06208	0.06208	0.03239	0.08638
0.45504	0.29979	0.13116	0.06156	0.06156	0.03212	0.08565
0.46405	0.30573	0.13376	0.06278	0.06278	0.03276	0.08735
0.44875	0.29564	0.12934	0.06071	0.06071	0.03168	0.08447
0.45127	0.29730	0.13007	0.06105	0.06105	0.03185	0.08494
0.44722	0.29464	0.12891	0.06051	0.06051	0.03157	0.08418
0.42354	0.27904	0.12208	0.05730	0.05730	0.02990	0.07973
0.45605	0.30046	0.13145	0.06170	0.06170	0.03219	0.08584
0.44768	0.29494	0.12904	0.06057	0.06057	0.03160	0.08427
0.46587	0.30693	0.13428	0.06303	0.06303	0.03289	0.08769
0.44835	0.29538	0.12923	0.06066	0.06066	0.03165	0.08439
0.45380	0.29898	0.13080	0.06140	0.06140	0.03203	0.08542
0.51838	0.34152	0.14942	0.07013	0.07013	0.03659	0.09758
0.51951	0.34227	0.14974	0.07029	0.07029	0.03667	0.09779
0.51749	0.34094	0.14916	0.07001	0.07001	0.03653	0.09741

0.53474	0.35230	0.15413	0.07235	0.07235	0.03775	0.10066
0.54181	0.35696	0.15617	0.07330	0.07330	0.03825	0.10199
0.54035	0.35600	0.15575	0.07311	0.07311	0.03814	0.10171
0.54194	0.35704	0.15621	0.07332	0.07332	0.03825	0.10201
0.55080	0.36288	0.15876	0.07452	0.07452	0.03888	0.10368
0.61889	0.40774	0.17838	0.08373	0.08373	0.04369	0.11650
0.62552	0.41211	0.18030	0.08463	0.08463	0.04415	0.11775
0.62333	0.41066	0.17967	0.08433	0.08433	0.04400	0.11733
0.60381	0.39780	0.17404	0.08169	0.08169	0.04262	0.11366
0.69472	0.45770	0.20024	0.09399	0.09399	0.04904	0.13077
0.73598	0.48488	0.21214	0.09957	0.09957	0.05195	0.13854
0.69430	0.45742	0.20012	0.09393	0.09393	0.04901	0.13069
0.69702	0.45921	0.20091	0.09430	0.09430	0.04920	0.13120
